# Coordination of cytoskeletal dynamics and cell behaviour during *Drosophila* abdominal morphogenesis

**DOI:** 10.1242/jcs.235325

**Published:** 2020-03-30

**Authors:** Pau Pulido Companys, Anneliese Norris, Marcus Bischoff

**Affiliations:** Biomedical Sciences Research Complex, University of St Andrews, North Haugh, St Andrews KY16 9ST, UK

**Keywords:** Actomyosin contractility, Apical constriction, Cell migration, *Drosophila*, Pulsed contractions

## Abstract

During morphogenesis, cells exhibit various behaviours, such as migration and constriction, which need to be coordinated. How this is achieved remains elusive. During morphogenesis of the *Drosophila* adult abdominal epidermis, larval epithelial cells (LECs) migrate directedly before constricting apically and undergoing apoptosis. Here, we study the mechanisms underlying the transition from migration to constriction. We show that LECs possess a pulsatile apical actomyosin network, and that a change in network polarity correlates with behavioural change. Exploring the properties of the contractile network, we find that cell contractility, as determined by myosin activity, has an impact on the behaviour of the network, as well as on cytoskeletal architecture and cell behaviour. Pulsed contractions occur only in cells with intermediate levels of contractility. Furthermore, increasing levels of the small Rho GTPase Rho1 disrupts pulsing, leading to cells that cycle between two states, characterised by a junctional cortical and an apicomedial actin network. Our results highlight that behavioural change relies on tightly controlled cellular contractility. Moreover, we show that constriction can occur without pulsing, raising questions why constricting cells pulse in some contexts but not in others.

## Abstract (precis)

**Summary:** During *Drosophila* morphogenesis, the larval epithelial cells transit from migration to constriction. A change in the polarity of their pulsatile cytoskeletal network and tightly controlled cellular contractility underlie this transition.

## INTRODUCTION

During development, morphogenetic processes ultimately shape the organism (Lecuit and Le Goff, 2007). Such processes are driven by various cell behaviours, e.g. intercalation (Bertet et al., 2004; Irvine and Wieschaus, 1994), division (Gong et al., 2004), migration (Montell, 1999) and shape change (Butler et al., 2009), all of which need to be coordinated (Lecuit and Le Goff, 2007). Little is known about how this coordination is achieved, how cells switch behaviour and how different behaviours occur simultaneously.

Cell behaviour is often directional. Planar cell polarity (PCP) directs cell behaviour in the plane of the tissue, coordinating, for example, junctional remodelling (Bosveld et al., 2012; Zallen and Wieschaus, 2004), division orientation (Baena-Lopez et al., 2005; Mao et al., 2011) and migration (Keller, 2002). Migrating cells have a protruding front and a contracting back (Lauffenburger and Horwitz, 1996), further indicating cytoskeletal polarity. In contrast, apically constricting cells show radial cell polarity (RCP) (Mason et al., 2013).

Ultimately, cell behaviour depends on the actin cytoskeleton. Cell migration relies on protrusive activity, such as lamellipodia formation (Lauffenburger and Horwitz, 1996). Cell shape changes, including apical constriction, which reduces apical cell area, depend on actomyosin contractility (Barrett et al., 1997; Kinoshita et al., 2008; Nikolaidou and Barrett, 2004; Sawyer et al., 2010). Increasing evidence suggests that the actin cytoskeleton shows rhythmical activity, such as actin flows during migration (Huang et al., 2013) and pulsed contractions, e.g. during junctional remodelling, neuroblast ingression, apical constriction, basal constriction and vertebrate neural tube closure (Christodoulou and Skourides, 2015; He et al., 2010; Martin et al., 2009; Rauzi et al., 2010; Roh-Johnson et al., 2012; Simoes et al., 2017; Solon et al., 2009). Pulsed contractions are driven by periodic actomyosin contractions (Blanchard et al., 2010; Levayer and Lecuit, 2012; Martin et al., 2009; Mason and Martin, 2011), which are thought to lead to cycles of cell area fluctuation, followed by stabilisation of the resulting smaller area (‘ratchet model’) (Martin et al., 2009).

Pulsed contractions are regulated by phosphorylation of Myosin II regulatory light chain [MRLC; Spaghetti squash (Sqh) in *Drosophila*] by Rho kinase (Rok) (Dawes-Hoang, 2005; Munjal et al., 2015; Vasquez et al., 2014), and its dephosphorylation by Myosin phosphatase (Fischer et al., 2014; Munjal et al., 2015; Valencia-Expósito et al., 2016). In addition, Rok inhibits the Myosin-binding subunit of the Myosin phosphatase complex (Mbs) (Kimura et al., 1996). Upstream of Rok, the small GTPase Rho1 is involved in regulating actomyosin contractility in many contexts, from rear retraction in migrating cells (Ridley, 2003) to pulsed contractions (Mason et al., 2016; Munjal et al., 2015). The activity of Rho1 is regulated by activating guanine nucleotide exchange factors (GEFs) and inhibitory GTPase-activating proteins (GAPs) (Graessl et al., 2017; Mason et al., 2016).

**Fig. 1.**
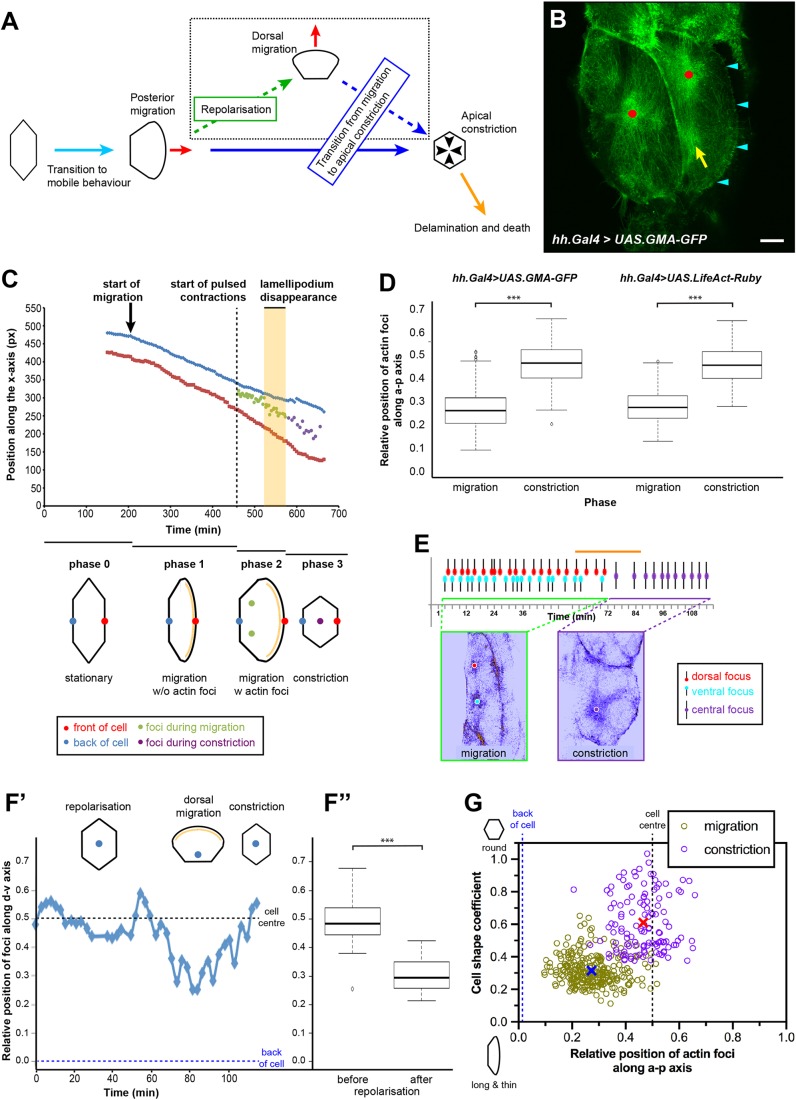
**LECs undergo pulsed contractions that correlate with their behaviour.** (A) LEC behaviour throughout morphogenesis (Bischoff, 2012). (B) Two LECs undergoing pulsed contractions. GMA-GFP labels F-actin. Left cell contracts; right cell migrates and contracts. Red dots, assembling actin foci; yellow arrow, disassembling actin focus; cyan arrowheads, lamellipodium. Note junctional actin pool. Scale bar: 10 µm. (C) LECs go through four phases of behaviour. Actin foci and front and back of cell were tracked over time. Foci are first positioned at back of cell, but once lamellipodium disappears (yellow area), foci move to cell centre. (D) Relative position of actin foci along a-p axis in GMA-GFP and LifeAct-Ruby pupae during migration and constriction. 0.5, cell centre; 0, cell back. GMA-GFP: *n *(migration)=278 foci in seven individual pupae; *n *(constriction)=123 foci in seven individual pupae. LifeAct-Ruby: *n *(migration)=198 foci in seven individual pupae; *n *(constriction)=123 foci in seven individual pupae; ***P<0.001. (E) Periodicity of actin foci. Two foci alternate during migration; single focus during constriction. Orange horizontal line indicates period of lamellipodium disappearance; here, pulsing is less regular. (F) Actin foci during dorsal migration after repolarisation. (F′) Relative position of focus along d-v axis, tracked over time. Focus moves towards new cell back, as defined by newly formed lamellipodium. (F″) After repolarisation, foci are positioned significantly more towards the ‘new’ cell back than before. *n *(before)=41 foci in three individual pupae, *n *(after)=39 foci in three individual pupae; ***P<0.001. (G) Cell shape versus relative position of actin foci along a-p axis during migration and constriction. Cells that are long along the d-v axis and thin along the a-p axis tend to show foci in back, whereas round cells tend to have one focus in centre. Blue cross, mean (migration); red cross, mean (constriction). Anterior, left; dorsal, top.

To gain insights into the mechanisms underlying the coordination of cell behaviour, we studied larval epithelial cells (LECs) during formation of the adult abdominal epidermis of *Drosophila*. During this process, the LECs are replaced by the adult histoblasts (Bischoff and Cseresnyes, 2009; Madhavan and Madhavan, 1980; Ninov et al., 2007). We have shown previously that LECs undergo directed migration followed by a transition to apical constriction, which eventually leads to delamination and apoptosis (Bischoff, 2012) (Fig. 1A). During migration, LECs form crescent-shaped lamellipodia and migrate posteriorly (Bischoff, 2012). LECs can only move if neighbours provide space by either migrating, reducing apical area or undergoing apoptosis (Bischoff, 2012) (Fig. S1A).

Studying the LEC cytoskeleton during abdominal morphogenesis, we show that LECs possess an apicomedial actomyosin network that undergoes pulsed contractions. The network is planar polarised during migration, undergoing pulsed contractions in the cell back, while the front protrudes. Contractions then re-localise to the cell centre, displaying radial polarity during constriction. Thus, behavioural change correlates with a change in the polarity of the contractile cytoskeletal network. To explore how manipulating actomyosin contractility affects the contractile network, we interfered with Rho1, Rok and Myosin phosphatase. We show that cellular contractility levels have an impact on the behaviour of the contractile network, with pulsed contractions only occurring in cells with intermediate contractility levels. We refer to ‘contractility’ as the ability of a cell to contract its actomyosin network (or increase stress in this network, if cell deformation is resisted), which is ultimately determined by its amount of active myosin II. Interestingly, increasing Rho1 levels interferes with pulsed contractions and leads instead to a cycling of cells between two states characterised by a junctional cortical and an apicomedial actin network. Thus, increasing contractility is sufficient to have an impact on cytoskeletal architecture and, consequently, cell behaviour. Additionally, we show that apical constriction can take place without pulsing, raising questions why constricting cells pulse in some contexts but not in others.

## RESULTS

### LECs undergo periodic apical contractions

To gain insights into the transition of LECs from migration to constriction, we analysed the behaviour of their actin cytoskeleton. We imaged LECs using *in vivo* 4D microscopy of the F-actin marker GMA-GFP, an actin-binding fragment of moesin fused with GFP (Bloor and Kiehart, 2001). GMA-GFP revealed a dynamic apicomedial actin network that contracted periodically (Fig. 1B; Fig. S1B). We observed ‘flows’, where fluorescence moved through the cell, and ‘foci’, where fluorescence coalesced in distinct regions (Fig. 1B; Fig. S2A′; Movie 1). Besides this dynamic pool of actin, GMA-GFP also labelled junctional cortical actin at cell–cell interfaces as well as persistent apicomedial actin bundles (Fig. 1B). We observed pulsed contractions throughout the epithelium, both in the anterior (A) and posterior (P) compartments (Fig. S1C). However, individual LEC behaviour varied in different regions of the epithelium, in particular with respect to cell shape (Fig. S1A) (Bischoff, 2012). To enable comparability, we thus focused our analysis on LECs in a particular region at the front of the P compartment (Fig. S1A).

### The activity of the pulsatile network correlates with LEC behaviour

Contractile behaviour correlated with four distinct phases of LEC behaviour (Fig. 1C; Movie 2): Phase 0: stationary LECs without visible cytoskeletal activity.

Phase 1: during early migration, LECs created a lamellipodium and migrated posteriorly, and the cytoskeleton showed diffuse apical activity.

Phase 2: during late migration, LECs produced a lamellipodium at the front and two actin foci in the back (Fig. 1B–E). The individual actin foci assembled with a period of 180±0.7 s (median±s.e.m.; *n*=276 foci in seven pupae) and contractility alternated between the two foci with around half their individual period (90±2.4 s; median±s.e.m.; *n*=137 foci in seven pupae). LECs migrated faster when showing pulsed contractions in phase 2 than during phase 1 (Fig. S2B).

Phase 3: constricting LECs showed one central actin focus (Fig. 1B–E) with a pulse period comparable to foci in migrating LECs (180±3.3 s; median±s.e.m; *n*=118 foci in seven pupae). The pulse period did not increase as constriction progressed (late phase 3; 180±0.4 s; median±s.e.m; *n*=132 foci in four pupae).

This cytoskeletal behaviour was not an artefact of GMA-GFP expression, as the actin marker LifeAct-Ruby (Hatan et al., 2011) allowed similar observations (Fig. 1D; Fig. S2C).

The transition between phases 2 and 3 was characterised by lamellipodium disappearance and loss of two actin foci, with one focus appearing in the cell centre (Fig. 1C,E). The transition was not sharp; for the duration of the transition, pulsing became more irregular (*n*=4/7 LECs; Fig. 1E) and actin foci and flow patterns changed (*n*=5/7 LECs; Fig. S2A″).

In dorsally repolarising LECs (Fig. 1A), the actin foci reorganised to the new cell back (Fig. 1F; Movie 3).

Cytoskeletal activity was not only correlated with cell behaviour, but also with cell shape. During migration, cells tended to be elongated along the dorsoventral (d-v) axis, with actin foci in the back, whereas during constriction cells were rounder, with a central actin focus (Fig. 1G).

Overall, pulsed contractions of the LEC actin cytoskeleton adopt a highly coordinated pattern, which correlates with cell behaviour.

### The activity of the pulsatile network correlates with changes in LEC apical area

**Fig. 2.**
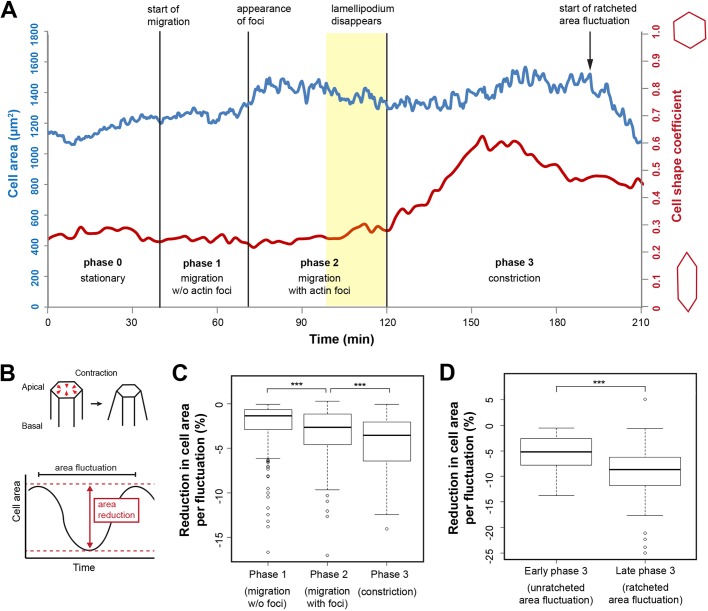
**LECs undergo cell area fluctuations that correlate with their behaviour.** (A) Tracking of LEC area over time. Cell area (blue) and cell shape coefficient (red) are shown. Extent of cell area fluctuations increases as cell goes through the four behavioural phases. Cell becomes rounder when lamellipodium disappears. Single cell shown. (B) Scheme depicting area reduction per fluctuation. (C) Reduction in cell area per area fluctuation in percent in phases 1, 2 and 3. *n *(phase 1)=379 fluctuations in seven LECs from individual pupae, *n *(phase 2)=223 fluctuations in seven LECs from individual pupae; *n *(phase 3)=149 fluctuations in seven LECs from individual pupae; ***P<0.001. (D) Reduction in cell area per area fluctuation during unratcheted versus ratcheted constriction. *n *(early)=74 fluctuations in four LECs from individual pupae, *n *(late)=69 fluctuations in four LECs from individual pupae; ***P<0.0011.

We next assessed the role of pulsed contractions in LEC behaviour. Pulsed contractions are crucial in driving apical constriction (Levayer and Lecuit, 2012; Martin et al., 2009; Mason and Martin, 2011; Solon et al., 2009). If this were the case in LECs, we would expect the rhythmical activity of actin foci to be correlated with apical cell area fluctuations as well as with an overall reduction in apical area. We therefore quantified apical area over time (Fig. 2A).

During early migration (phase 1), no foci were visible, but apical cell area was fluctuating slightly (Fig. 2B,C; Fig. S3A). Cells were elongated along the d-v axis (Fig. 2A; Fig. S3B). With the appearance of foci (phase 2), fluctuations increased significantly (Fig. 2C; Fig. S3A). Although cell shape varied considerably between individual cells, cells generally tended to become rounder (Fig. S3B) without reducing area (Fig. S3C′). During constriction (phase 3), area fluctuations increased further (Fig. 2C; Fig. S3A) and rounding continued (Fig. S3B). To become rounder, cells mainly changed shape along the d-v axis (Fig. S3D). Constriction can be divided into an ‘early’ phase, in which the apical area was not reduced (Fig. S3C″), and a ‘late’ phase, in which apical area decreased by ratcheted constrictions (Fig. 2A), which ultimately led to delamination. This ‘late’ constriction was characterised by a further significant increase in apical area fluctuation (Fig. 2D).

This sequence of behavioural changes resembles embryonic cells during gastrulation, which go through comparable phases of unconstricting, unratcheted and ratcheted contractions (Roh-Johnson et al., 2012; Xie and Martin, 2015). In LECs, however, unconstricting area fluctuations take place without detectable pulsatile behaviour, and all observed pulsed contractions lead to area fluctuations. Similar to unratcheting contractions (Roh-Johnson et al., 2012; Xie and Martin, 2015), during migration and early constriction, pulsed contractions of LECs did not necessarily result in an overall reduction of their apical area (Fig. 2A; Fig. S3C).

### Pulsed contractions drive apical area fluctuation

**Fig. 3.**
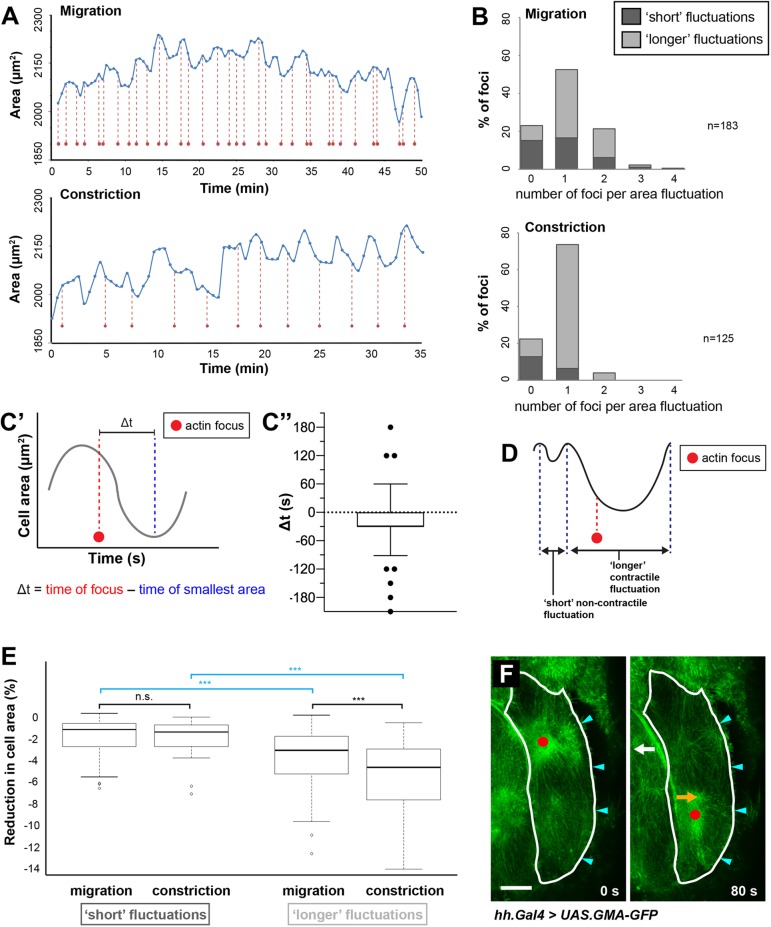
**Reduction in LEC area correlates with occurrence of actin foci.** (A) Cell area fluctuation and actin foci (red dots) for one LEC shown over time. During constriction, foci appear more regularly, one focus roughly corresponding with one fluctuation. (B) Number of actin foci per area fluctuation. Bars are subdivided depending on duration of fluctuation – ‘short' (≤90s) and ‘longer' fluctuations (>90s). Migrating LECs (top) show higher variability in foci number per fluctuation compared to constricting LECs (bottom). Migrating cells also show higher proportion of ‘short' fluctuations. Most fluctuations without foci are ‘short’ in both migrating and constricting LECs. (C) Analysis of time difference between occurrence of actin focus and smallest LEC area (Δt). (C′) Scheme depicting Δt. (C″) Plot showing Δt. *n*=118 foci in four individual pupae; box plot with 5th percentile, first quartile (Q1), median, third quartile (Q3) and 95th percentile, one outlier (–400) not shown. (D) Scheme illustrating ‘short’ non-contractile and ‘longer’ contractile fluctuation. (E) Reduction in cell area per pulsed contraction during migration and constriction, for ‘short’ and ‘longer’ fluctuations. Short fluctuations: *n *(migration)=70 fluctuations in seven LECs in individual pupae, *n *(constriction)=24 fluctuations in seven LECs in individual pupae; longer fluctuations: *n *(migration)=108 fluctuations in seven LECs in individual pupae, *n *(constriction)=101 fluctuations in seven LECs in individual pupae; ***P<0.001. (F) Migrating LECs constrict asymmetrically. Migrating LEC undergoing two consecutive contractions shown. GMA-GFP labels F-actin. Red dot, actin focus; cyan arrowheads, lamellipodium. Cell outline drawn at 0 s and the same outline shown at 80 s. At 80 s, cell expands in region of the focus present at 0 s (white arrow), whereas it contracts in region of the new focus (orange arrow). Scale bar: 10 µm.

We next asked whether pulsed contractions correlate with cell area fluctuations (Fig. 3A). We found that, in 51% of fluctuations in migrating LECs and in 74% of fluctuations in constricting LECs, one actin focus occurred during one fluctuation (Fig. 3B). We also found that foci appeared around 30±5.3 s (mean±s.e.m.) before LEC area was smallest (Fig. 3C). This shows that area fluctuations correlate with actin foci and suggests that the contractile event drives cell area reduction.

Besides area fluctuations that correlated with foci, we also observed fluctuations without foci (Fig. 3B). We hypothesised that these fluctuations might be due to external forces exerted by contracting neighbouring cells, the pulling by which could deform cells in addition to regular fluctuations. Such events might have a shorter duration and lead to smaller area reduction than pulsed contractions (Fig. 3D). Categorising individual fluctuations by duration, we found that the majority of fluctuations not coinciding with a focus were ‘short’ (≤90 s) (Fig. 3B). These ‘short’ fluctuations did not reduce the cell area much – the reduction, both during migration and constriction (Fig. 3E), was comparable to that in LECs that migrated without visible pulsatile activity (*P*=0.64, Kruskal–Wallis rank sum test with *post-hoc* test: χ^2^=0.9, d.f.=1). However, for ‘longer’ fluctuations (>90 s), there was a significant difference in area reduction per fluctuation between migration and constriction (Fig. 3E). Overall, this suggests that the majority of area fluctuations that occur without an accompanying actin focus are ‘short’ non-contractile fluctuations that might be due to pulling/pushing by neighbouring LECs.

Furthermore, in migrating LECs, the correlation between area fluctuations and actin foci was less strong than in constricting LECs; around 25% of the fluctuations in migrating LECs showed two foci, and overall the number of ‘short’ fluctuations involving foci was higher than in constricting LECs (Fig. 3B). The weaker correlation could be due to the two alternating contractile events in different cell regions affecting cell shape change unevenly (Fig. 3F). Moreover, area fluctuation could be reduced due to the cell's protrusive activity, as lamellipodia stabilise cell-cell interfaces (Movie 1).

Taken together, our observations suggest that the contractile apicomedial network reduces LEC area during each pulsed contraction leading to cell area fluctuation.

### LECs show distinct cytoskeletal architecture during migration and constriction

**Fig. 4.**
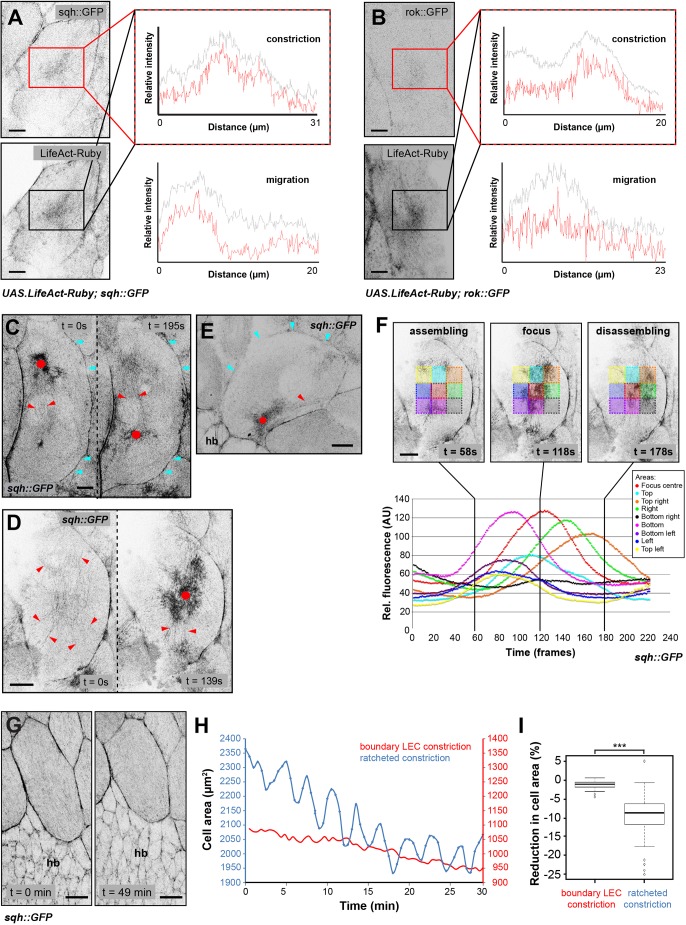
**Dynamic behaviour of the LEC cytoskeleton.** (A,B) LifeAct-Ruby co-localises with (A) Sqh::GFP and (B) Rok::GFP in actin foci and cell–cell interfaces, during migration and constriction. Plot profiles of relative fluorescence intensity in rectangular region of 20 µm^2^ shown. This function averages pixel intensities along the *y*-axis of the selected region before measuring intensities along the resulting line. (C–E) Sqh::GFP labels actin foci (dots), actin bundles (red arrowheads) and cell–cell interfaces. Cyan arrowheads, lamellipodium. (C) Posteriorly migrating LEC, showing actin focus and bundles in back as well as lamellipodium in front. Bundles are organised along d-v axis. (D) Constricting LEC before (left) and during (right) pulsed contraction. Actin bundles are organised radially. (E) Dorsally migrating LEC, showing actin focus and bundles in back and lamellipodium at front. (F) Analysis of actin focus coalescence. For the nine squares in the cell centre, plot profiles were used to quantify relative fluorescence intensity (arbitrary units, AU). Plot shows relative fluorescence for the nine squares over time. Black lines indicate time points of depicted images. Fluorescence first passes through peripheral squares before reaching centre. When focus disassembles, fluorescence moves back to periphery. (G–I) Constriction of boundary LECs during early morphogenesis. (G) Sqh::GFP labels cell–cell interfaces. Also, some diffuse labelling all over apical cell area, but no actin foci visible. LEC constricts over time. (H) Comparison of area fluctuations over time between LEC undergoing ratcheted constriction and constricting boundary LEC, which shows little area fluctuation. Note that boundary cell constricts rather slowly. (I) Reduction in cell area per pulsed contraction during ratcheted constriction and early boundary LEC constriction [*n *(boundary LECs)=39 area fluctuations in four LECs in individual pupae, *n *(ratched LECs)=69 fluctuations in four LECs in individual pupae; ***P<0.001]. All scale bars: 10 µm. hb, histoblasts. Anterior, left; dorsal, top.

Studying the apicomedial network further, we found that both Sqh::GFP (Royou et al., 2004) and Rok::GFP (Abreu-Blanco et al., 2014) colocalised with foci labelled with LifeAct-Ruby (Fig. 4A,B). This corroborates the notion that network contractility is created by actomyosin activity.

Sqh::GFP, Rok::GFP, LifeAct-Ruby and GMA-GFP not only labelled foci, but also localised to the cell cortex at junctional interfaces (Figs 1B and 4A,B). In early stationary and migratory LECs, Sqh::GFP levels are higher at anterior–posterior (a-p) interfaces, compared with d-v interfaces (Fig. S4A′,B). Constricting cells, however, showed no differences in Sqh::GFP localisation between a-p and d-v interfaces (Fig. S4A″,B).

**Fig. 5.**
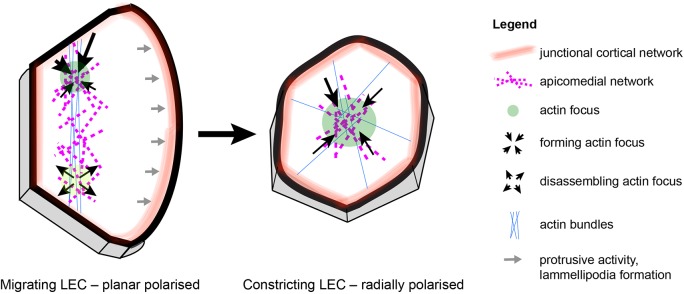
**LECs undergo complex changes in cytoskeletal architecture during their behavioural switch from migration to constriction.** Left: the cytoskeleton of a migrating LEC shows numerous features that indicate planar polarity: (1) protrusive activity in the lamellipodium; (2) a contractile apicomedial network in the back of the cell; (3) actin bundles that are oriented along the d-v axis in the back of the cell; (4) a planar polarised cortical network, which might contribute to contractility; and (5) a basolateral contractile flow in the back of the cell. Right: the cytoskeleton of a constricting LEC has a different architecture, which appears radially polarized: (1) a contractile medio-apical network in the centre of the cell; (2) actin bundles that are oriented radially; and (3) a cortical network, which localises around the whole cell and which might contribute to contractility. Anterior, left; dorsal, top.

In addition, Sqh::GFP and GMA-GFP also labelled actin bundles. Migrating LECs displayed actin bundles in their back, where also myosin and actin foci occurred (Fig. 4C; Fig. S4C,D). These bundles resembled stress fibres in migrating fibroblasts (Pellegrin and Mellor, 2007). Deformation of cells where actin bundles meet the cell–cell junctions suggests that the bundles can exert pulling forces (Fig. S4E). In constricting LECs, actin bundles appeared to connect a persistent contractile apicomedial network radially to the junctions (Fig. 4D; Movie 4). After repolarisation, actin bundles, like foci, were found in the new back of the dorsally migrating cells (Fig. 4E). Thus, besides foci localisation, other aspects of cytoskeletal architecture also correlate with cell behaviour and polarity, which changes as cells transit from migration to constriction (Fig. 5).

### Actin foci assembly begins in the periphery of the apicomedial network

The large size of the LECs allowed us to study the assembly and disassembly of foci in detail using Sqh::GFP. We defined nine sectors that covered the central area of constricting LECs and measured average fluorescence over time. In 91% of analysed contractions, foci began to assemble in the periphery of the apicomedial network (*n*=65 contractions in 10 LECs in nine pupae). The fluorescence signal then coalesced in the cell centre, before dissipating towards the periphery (Fig. 4F; Movie 4).

### Not all LECs undergo pulsed contractions

Studying Sqh::GFP dynamics, we noticed that those LECs which border histoblast nests at the beginning of morphogenesis (boundary LECs; Teng et al., 2017), constrict apically without displaying actin foci. Instead, they show Sqh::GFP at their cell–cell junctions, as well as diffuse labelling across their apical surface (Fig. 4G; Movie 5). When constricting, boundary LECs showed little apical area fluctuations (Fig. 4H,I). This highlights that not all LECs that reduce their apical area do so while contracting in a pulsatile manner.

### Reducing actomyosin contractility by interfering with myosin II phosphorylation

To gain insights into the regulation of pulsed contractions, we interfered genetically with Rok and the Myosin-binding subunit (Mbs) of Myosin phosphatase. From previous studies, we expected both RNAi knockdown of Rok and constitutive activation of Mbs to reduce the amount of phosphorylated Myosin II and thus cell contractility (Dawes-Hoang, 2005; Fischer et al., 2014; Lee and Treisman, 2004; Munjal et al., 2015; Valencia-Expósito et al., 2016; Vasquez et al., 2014). In LECs, over-expression of a constitutively active form of Mbs [MbsN300 (Lee and Treisman, 2004)] impairs apical constriction (Ninov et al., 2007).

**Fig. 6.**
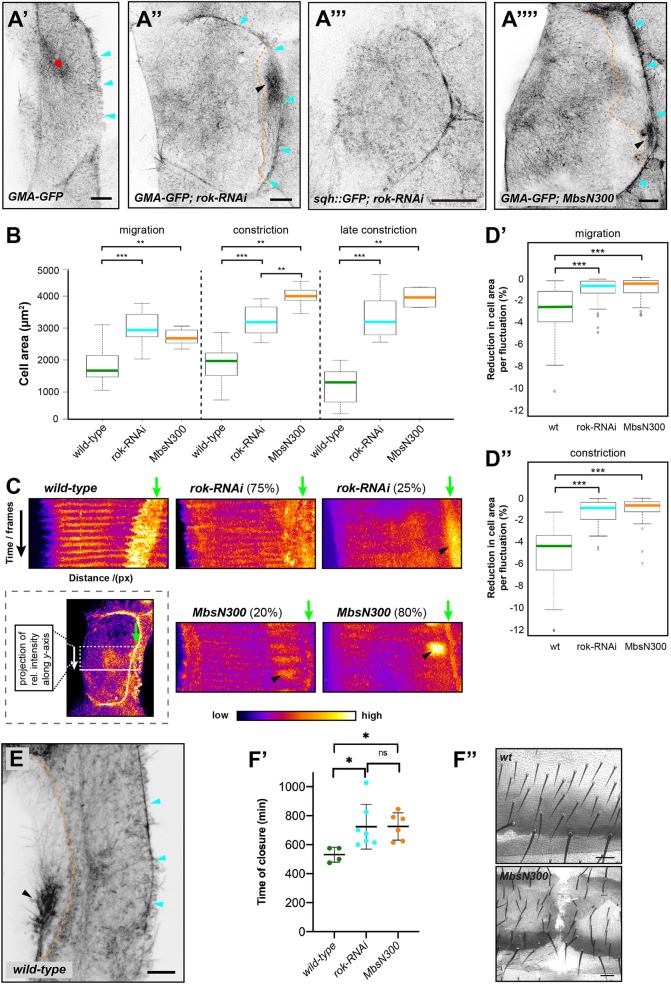
**Reduction in LEC contractility interferes with actin foci formation, cell shape and area fluctuation.** (A) Control (A′), *rok-RNAi* (A″) and *MbsN300* (A‴′) LECs during migration. GMA-GFP labels F-actin. Cells generate lamellipodium (cyan arrowheads). Wild-type LEC shows actin focus (red dot), whereas *rok-RNAi* and *MbsN300* LECs show more diffuse cytoskeleton labelling without foci. Neighbours create contractile flows in their back (black arrowheads, dotted orange line outlines overlap between cells). Scale bars: 10 µm. (A‴) *rok-RNAi* LEC labelled with Sqh::GFP constricts without focus formation. Cell only shows diffuse Sqh::GFP. (B) Apical area of wild-type (wt), *rok-RNAi* and *MbsN300* LECs during migration, early constriction and late constriction. Migration and constriction: *n *(wt)=14 LECs, *n *(*rok-RNAi*)=8 LECs, *n *(*MbsN300*)=5 LECs; late constriction: *n *(wt)=13 LECs, *n *(*rok-RNAi*)=8 LECs, *n *(*MbsN300*)=4 LECs; **P<0.01, ***P<0.001. (C) Kymograph of LEC cell centre during constriction. Kymograph shows *y*-projection of rectangular region of interest (see scheme in box) over time. GMA-GFP labels F-actin. Wild-type LEC shows rhythmic actin activity. Due to area fluctuation, membrane region appears broad (green arrow). In 75% of *rok-RNAi* and 20% of *MbsN300* LECs, some rhythmical activity visible, but more diffuse than wild-type. In 25% of *rok-RNAi* and 80% of *MbsN300* LECs, no rhythmical activity visible. Contractile flows in back of neighbouring cell visible (black arrowhead). In mutants, membrane region appears thin due to fewer area fluctuations (green arrows). (D) Reduction in cell area per pulsed contraction in wild type, *rok-RNAi* and *MbsN300*. ***P<0.001. (D′) Migrating LECs. *n *(wt)=48 fluctuations in four LECs in individual pupae, *n *(*rok-RNAi*)=65 fluctuations in five LECs in individual pupae, *n *(*MbsN300*)=51 fluctuations in four LECs in individual pupae. (D″) Constricting LECs. *n *(wt)=39 fluctuations in four LECs in individual pupae, *n* (*rok-RNAi*)=67 fluctuations in five LECs in individual pupae, *n *(*MbsN300*)=48 fluctuations in three LECs in individual pupae. (E) Migrating wild-type LEC creates contractile flow in its back (black arrowhead). GMA-GFP labels F-actin. Scale bar: 10 µm. (F) *rok-RNAi* and *MbsN300* prolong time of abdominal closure leading occasionally to dorsal cleft phenotype. (F′) Timing of abdominal closure. Wild type, *rok-RNAi* and *MbsN300* shown. *n *(*wt*)=4 pupae, *n *(*rok-RNAi*)=7, *n *(*MbsN300*)=6 pupae; individual data points and means±s.d. shown; *P<0.05. (F″) Wild-type cuticle and dorsal cleft phenotype in MbsN300 cuticle. Scale bars: 50 µm.

Rok-RNAi did not interfere with LEC migration (Fig. S5A,B), but LECs showed a phenotype indicative of reduced contractility. Firstly, LECs had an increased apical area compared with controls, both during migration and constriction (Fig. 6A′,A″,B). An increased apical area suggests reduced contractility in the apicomedial network due to insufficient levels of active myosin to keep network tension and, thus, maintain cell size. In amnioserosa cells, reducing contractility also increased cell area (Fischer et al., 2014). Secondly, Rok-RNAi affected pulsed contractions. Six out of eight analysed LECs showed some pulsatile activity, whereas in two out of eight LECs, foci were absent (Fig. 6C). Where foci could be observed, their pulsation period was comparable to wild type (180±1.7 s; median±s.e.m.; *n*=182 foci in five pupae; *P*=0.11, Kruskal–Wallis H test with *post-hoc* χ^2^=2.59, d.f.=1), but foci were more diffuse (Fig. 6C; Movie 6). Where foci were absent, GMA-GFP labelled a not very dynamic apicomedial network, which did not generate any foci and only showed some diffuse activity (Fig. 6A″; Movie 7). We found a comparable phenotype using Sqh::GFP as a marker; in 58% of pupae, LECs showed only diffuse activity and no foci (*n*=24; Fig. 6A‴; Movie 8). Thirdly, apical area fluctuations were reduced, both during migration and constriction (Fig. 6D; Fig. S6A). During constriction, fluctuations (–0.9±0.1% of cell area; median±s.e.m.; *n*=67 fluctuations in five pupae) were even smaller than in early migrating wild-type LECs, which do not show pulsed contractions (−1.4±0.1% of cell area; median±s.e.m.; *n*=379 fluctuations in seven pupae; *P*<0.001, Kruskal–Wallis H test with *post-hoc* χ^2^=64.29, d.f.=1). A reduction in the ability to generate foci as well as apical area fluctuations suggests insufficient levels of active myosin to generate pulsed contractions, which can deform the cell.

In addition, we observed that, in the back of migrating cells, actin accumulated periodically, travelling along the membrane in a d-v direction (Fig. 6A″,C; Movies 6 and 7). These contractile flows only occurred basolaterally underneath protruding lamellipodia of neighbouring cells. Occasionally, we also observed this behaviour in wild-type LECs (Fig. 6E). These flows might be a reaction of the cells to forces created by the lamellipodia of their neighbours (Bischoff and Cseresnyes, 2009). Rok-RNAi might increase these flows, as due to their reduced contractility the cells can be deformed more easily by the lamellipodia of their neighbours. Force-dependent Myosin II accumulation has been observed in *Drosophila* embryos (Bailles et al., 2019; Fernandez-Gonzalez et al., 2009) and during myoblast fusion (Kim et al., 2015).

Over-expression of MbsN300 showed a similar phenotype to Rok-RNAi (Fig. 6A‴′–D; Figs S5C and S6A; Movies 9 and 10). However, the MbsN300 phenotype was stronger, with a higher proportion of ‘strong’ phenotypes, which did not generate any actin foci (Fig. 6C).

To investigate whether defects in LEC contractility have an impact on morphogenesis, we assessed the time of abdominal closure in control, Rok-RNAi and MbsN300 pupae. We found that in both experiments, closure was delayed (Fig. 6F′). In some cases, this led to closure defects, where LECs did not complete morphogenesis successfully [2.6% of Rok-RNAi pupae (*n*=39); 60.8% of MbsN300 pupae (*n*=51); Fig. 6F″]. This suggests that impaired contractility can lead to morphogenesis defects, but that in many cases, LECs still delaminate.

Our data show that activity of both kinase and phosphatase (which activate and deactivate myosin II, respectively) is crucial for cell contractility, in particular for the formation of pulsed contractions. Interestingly, even without detectable actin foci and thus significantly less apical area fluctuation, LECs can constrict and delaminate successfully.

### Increasing actomyosin contractility by constitutive activation of Rok and Rho1

Next, we asked what effect an increase in contractility has on LEC pulsatile activity. In the *Drosophila* embryo, constitutive activation of Sqh or Rho1 interferes with the activity of the pulsatile actomyosin network (Fischer et al., 2014; Mason et al., 2016; Munjal et al., 2015; Valencia-Expósito et al., 2016; Vasquez et al., 2014).

**Fig. 7.**
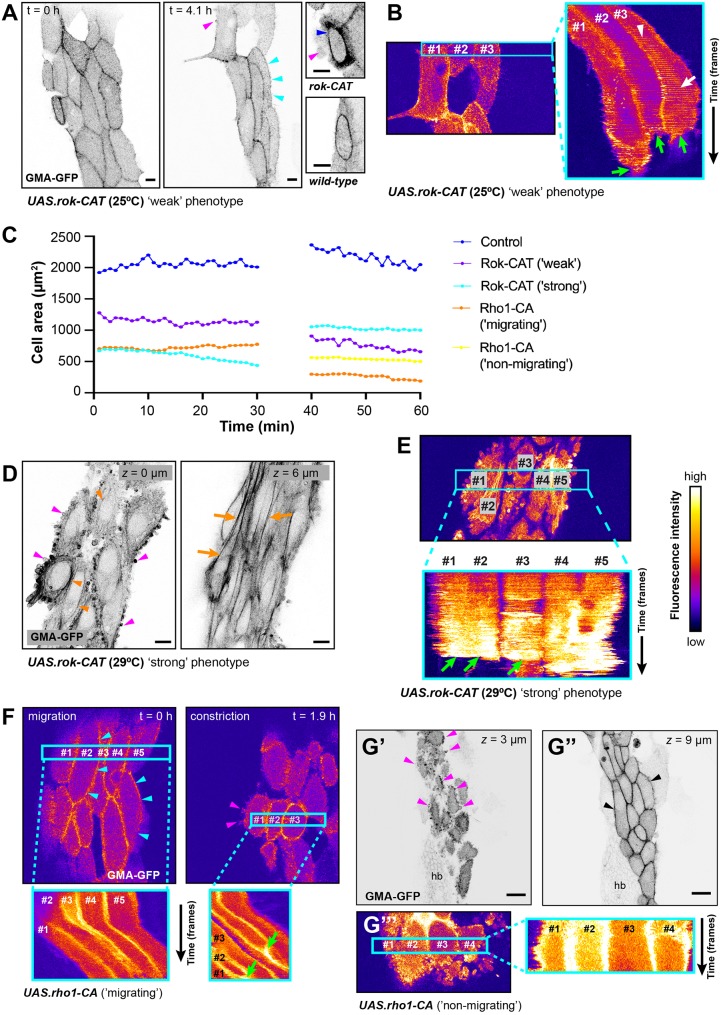
**Increasing LEC contractility by expression of constitutively active Rok (Rok-CAT) and Rho1 (Rho-CA).** GMA-GFP labels F-actin. All scale bars: 10 µm. (A,B) ‘Weak’ Rok-CAT phenotype; transgene expression at 25°C. (A) Start of morphogenesis (left) and LEC migration (right). LECs migrate, showing some blebbing (pink arrowheads) and lamellipodia (cyan arrowheads). Detailed images: compared with controls, constricting Rok-CAT cell shows high cortical actin (blue arrowhead) and blebbing. (B) Kymograph of three LECs (right-hand panel). Projected region indicated in left panel. Cells move to the right, eventually cease to move, constrict and delaminate (green arrows). In cell number 3, contractile activity is in back during migration (white arrowhead) and in centre during constriction (white arrow). (C) Cell area over time for control, ‘weak’ and ‘strong’ Rok-CAT as well as ‘migrating’ and ‘non-migrating’ Rho1-CA LECs. Part of migration and constriction phase of representative cells shown. The control LECs as well as the ‘weak’ Rok-CAT LECs show cell area fluctuation. The ‘strong’ Rok-CAT and both Rho-CA cells display no area fluctuation. *n* (wt)=4 LECs in individual pupae; *n *(*rho1-CA*, *rok-CAT*)=3 LECs in individual pupae for each phenotype. (D,E) ‘Strong’ Rok-CAT phenotype; transgene expression at 29°C. (D) Constricting Rok-CAT LECs. Left: apically, LECs show extensive blebbing (pink arrowheads) and cortical actin bundles (orange arrowheads). Right: more basally, cells generate extensive stress fibre-like actin bundles (orange arrows). (E) Kymograph of five Rok-CAT LECs (bottom panel). Projected region indicated in top panel. No rhythmical cytoskeletal activity. Cells eventually delaminate (green arrows). (F) Rho1-CA, ‘migrating’ phenotype. Top left: migrating LECs showing cortical actin and lamellipodia (cyan arrowheads). Top right: constricting LECs showing cortical actin and blebbing (pink arrowheads). Bottom: kymographs of LECs during migration and constriction. No rhythmical cytoskeletal activity visible. Projected region indicated by cyan boxes. Eventually cells constrict and delaminate (green arrowheads). (G) Rho1-CA, ‘non-migrating’ phenotype. Micrographs of apical (G′) and lateral (G″) *z*-section. Constricting cells show extensive apical cortical blebbing (pink arrowheads) and actin labelling at cell interfaces (black arrowheads). Scale bars: 20 µm. (G‴) Kymograph of four LECs. Projected region indicated by cyan box. No rhythmical cytoskeletal activity visible.

Firstly, we over-expressed in LECs a constitutively active form of Rok (Rok-CAT), which leads to excessive phosphorylation and thus activation of myosin II (Verdier et al., 2006; Warner and Longmore, 2009). Using Gal80ts to repress Rok-CAT expression before pupal stages allowed us to assess different levels of Rok-CAT expression by inactivating the repressor either efficiently at 29°C for strong expression or less efficiently at 25°C for weaker expression. Gal80ts expression did not interfere with pulsed contractions in controls (Fig. S6B). This approach resulted in two distinct phenotypes, as follows. (1) In ‘weak’ phenotypes, LECs showed migratory and constrictive behaviour (Fig. 7A,B; Movie 11; 100% of 25°C experiments, *n*=5 pupae; 35.3% of 29°C experiments, *n*=17 pupae). These two phenotypes were consistent with the differences in expression levels of the transgene expected due to the temperature used to inhibit Gal80ts repression. As in the wild type, migrating LECs displayed periodic actin foci in their back, which moved to the centre during constriction (Fig. 7B) and led to area fluctuations (Fig. 7C). Also, LECs underwent delamination, which was characterised by a more pronounced contractile ring compared with wild type (Fig. 7A; Movie 11). Furthermore, constricting cells showed blebbing at the apical junctional cortex (Fig. 7A; Movie 11). (2) In ‘strong’ phenotypes, LECs did not migrate and only constricted (Fig. 7D,E; Movie 12; 64.7% of 29°C experiments, *n*=17 pupae). Constricting cells showed extensive blebbing and formed cortical actin bundles apically, as well as stress-fibre-like bundles more basally (Fig. 7D). In addition, cells did not show pulsatile activity (Fig. 7E) and lacked notable area fluctuation (Fig. 7C).

Secondly, we over-expressed in LECs a constitutively active form of Rho1 (Rho1-CA; Fanto et al., 2000). We have shown previously that Rho1-CA over-expression leads to loss of migration, forcing LECs to only undergo apical constriction (Bischoff, 2012). We hypothesised that over-expressing Rho1-CA should lead to more Rok being activated and thus a phenotype similar to Rok-CAT. Indeed, Rho1-CA phenotypes resembled Rok-CAT phenotypes, with cells displaying cortical blebbing, junctional cortical actin localisation and absence of rhythmic cytoskeletal activity (Fig. 7F,G; Movies 13 and 14), which is reflected in the lack of cell area fluctuation (Fig. 7C). However, unlike Rok-CAT cells, Rho-CA LECs did not create excessive cortical actin bundles (Fig. 7F,G). With respect to cell migration, Rho1-CA pupae showed two phenotypes: ‘migrating’ phenotypes (29%, *n*=7 pupae; Fig. 7F; Movie 13), and ‘non-migrating’ phenotypes (71%), in which LECs only constricted (Fig. 7G; Movie 14). Unlike ‘weak’ Rok-CAT cells, in none of the analysed Rho-CA pupae did cells show pulsed contractions (*n*=7 pupae).

These data show that increasing contractility interferes with pulsed contractions and apical area fluctuations. Expressing Rho-CA in all LECs results in abdominal cleft phenotypes in 63% of cases (Bischoff, 2012). Thus, although apical constriction is impaired, LECs delaminate successfully in many cases.

### Increasing levels of Rho1 induces rhythmical remodelling of the apicomedial network

**Fig. 8.**
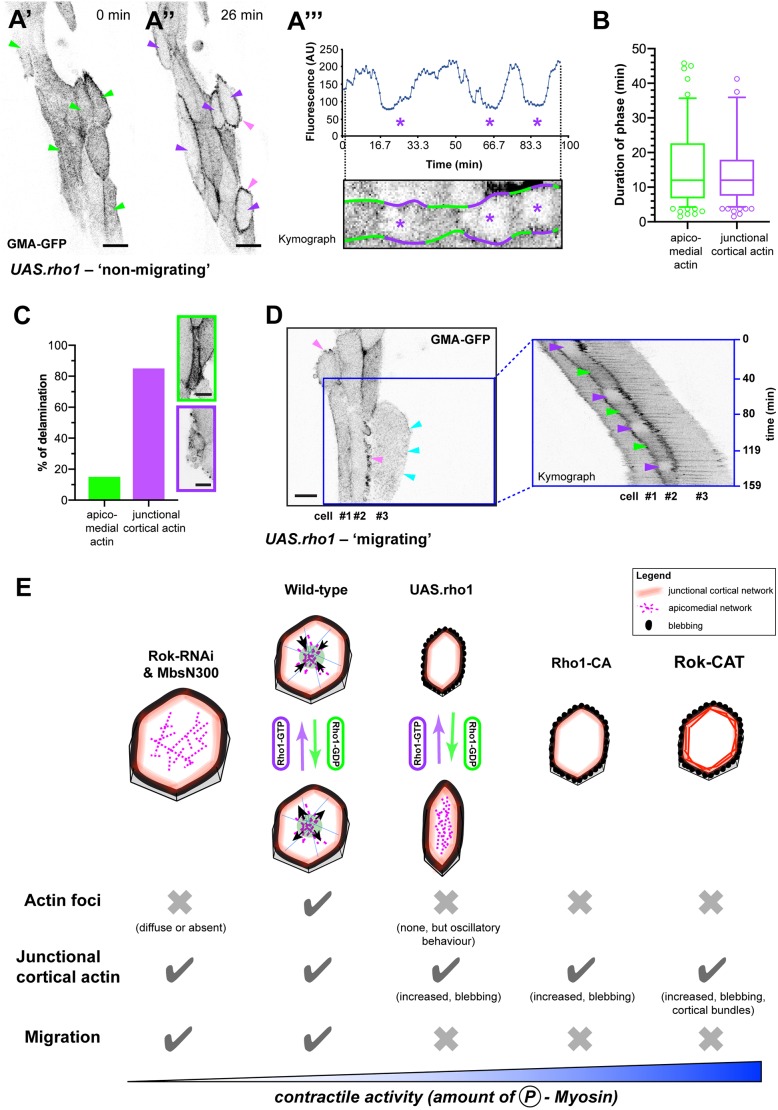
**Over-expression of Rho1 leads to oscillatory changes in the cytoskeletal network.** (A) ‘Non-migrating’ phenotype. GMA-GFP labels F-actin. Scale bar: 20 µm. LECs cycle between two states: (A′) presence of apicomedial actin (green arrowheads) and (A″) absence of apicomedial actin but increased junctional cortical actin (purple arrowheads) and cortical blebbing (pink arrowheads). (A‴) Fluorescence intensity of cell centre of cycling LEC (top) and kymograph of same cell (bottom). Purple asterisks indicate junctional cortical states. (B) Duration of the two states. *n*=86 states in 30 cells in five pupae; box plot with 10th percentile, first quartile, median, third quartile and 90th percentile. *P*=0.9677. (C) LECs delaminate in both states. Top box, cell delaminating with apicomedial actin and no blebbing; note that actin is increased. Bottom box, cell delaminating with junctional cortical actin and blebbing. Scale bars: 10 µm (*n*=33 LECs in five pupae). (D) ‘Migrating’ phenotype. GMA-GFP labels F-actin. Scale bar: 20 µm. Left: LECs forming lamellipodia (cyan arrowheads) and blebs (pink arrowheads). Right: kymograph from area indicated by blue box. Cells migrate posteriorly. Cell number 2 cycles between apicomedial (green arrowheads) and junctional cortical actin state (purple arrowheads). (E) Cellular contractility coordinates LEC behaviour and cytoskeletal dynamics. Amount of LEC contractility affects presence of pulsed contractions (actin foci), amount of junctional actin, as well as presence or absence of cortical blebbing, and migration. Only with intermediate contractility levels, cells show pulsed contractions. LECs with increased Rho1 levels (*UAS.rho1*) cycle between two distinct states, which do not show pulsed contractions. Reducing contractility (Rok-RNAi, MbsN300) interferes with pulsations and leads to larger cell area. Increasing contractility (Rho-CA) leads to cortical blebbing and interferes with pulsations and migration; F-actin localises to junctional cortex. Very strong increase of contractility (Rok-CAT) additionally leads to formation of cortical actin bundles.

Besides activating contractility by expressing constitutively active forms of Rok and Rho1, we also increased the amount of wild-type Rho1 in LECs (*UAS.rho1*; Prokopenko et al., 1999). Interestingly, these cells did not undergo pulsed contractions, but cycled between two distinct states characterised by (1) the presence of apicomedial actin and (2) the absence of apicomedial actin but increased junctional cortical actin and blebbing (Fig. 8A; Movie 15; *n*=110 switches in 30 cells in five pupae). There was no difference in the mean duration of each state; however, there was considerable variation (Fig. 8B). Cells switched states up to 11 times in 6.5 h (on average 4±2.5 times in 3±2.2 h; mean±s.d.). Interestingly, cells delaminated in either state, although most cells delaminated in the junctional cortical state (Fig. 8C). As well as affecting the apicomedial network, Rho1 over-expression also affected LEC migration; in 40% of the pupae (*n*=5), LECs did not migrate (‘non-migrating’ phenotype; Fig. 8A; Movie 15; ‘migrating phenotype’; Fig. 8D).

Our results suggest that adding additional Rho1, which can be switched on and off by the endogenous machinery of the cells, causes LECs to cycle between two states characterised by distinct cytoskeletal networks. Thus, rhythmical activity does not appear to be limited to pulsed contractions, but also extends to more general rhythmical remodelling of cytoskeletal architecture (Fig. 8E).

## DISCUSSION

We show that the apicomedial actomyosin network of LECs undergoes pulsed contractions, while cells transit from migration to constriction (Fig. 1A,B). During this transition, cytoskeletal activity is highly coordinated and correlates with cell behaviour (Fig. 1B–G). Pulsed contractions are correlated with cell area fluctuations (Figs 2 and 3).

We find that a cell's level of contractility determines the behaviour of its actin cytoskeleton, with pulsed contractions depending on intermediate levels of contractility, as well as cytoskeletal architecture and cell behaviour (Fig. 8E). Moderately increasing contractility causes LECs to cycle between two states, characterised by a junctional cortical and an apicomedial actin network, respectively (Fig. 8A–D). Moreover, our data suggest that apical constriction can occur without pulsed contractions, raising questions why constricting cells pulse in some contexts but not in others.

### The cytoskeleton of LECs undergoes dynamic change

During migration, LECs are planar polarised. They protrude at the front and undergo pulsed contractions in the back (Fig. 1B–E), where they also create d-v oriented actin bundles (Fig. 4C; Fig. S4C,D). In their junctional cortical network, Sqh::GFP localises preferentially to the a-p junctions (Fig. S4A′,B), similar to embryonic cells during germband extension (Zallen and Wieschaus, 2004). As LECs constrict, their apicomedial network becomes radially polarised, with a central actin focus and radial actin bundles that connect the network to the junctions, resembling a spider's web (Figs 1B–E and 4D). In addition, Sqh::GFP is distributed evenly across all junctions (Fig. S4A″,B). Thus, LEC behavioural change is accompanied by a change in cell polarity from PCP to RCP that underlies a change in cytoskeletal activity as well as an overall reorganisation of cytoskeletal architecture (Fig. 5).

The polarity of LEC migration depends on PCP signalling (Arata et al., 2017; Bischoff, 2012), but little is known about the link between PCP and cytoskeletal asymmetry (Arata et al., 2017). Cytoskeletal asymmetry could be due to localised activity of small Rho GTPases, with Rho1 controlling pulsed contractions in the cell back and Rac1 promoting lamellipodia formation at the front. Such mutually exclusive localisation has been shown in various cell types (Cao et al., 2015; Ohta et al., 2006; Sander et al., 1999). How RCP is established in constricting LECs is still unknown.

### Generation of pulsatile activity in LECs

LECs create very large actin foci when contracting, but their foci period is comparable to other systems that create smaller foci (reviewed in Gorfinkiel and Blanchard, 2011). A possible explanation for the short period of the large foci is that foci in LECs are initiated within a large area in the periphery of the cell (Fig. 4F) and not in a small region from which the contractile event then expands (which would potentially slow down foci formation). Focus formation involves both the recruitment of novel actin and Sqh as well as advection (Munjal et al., 2015), where already recruited actin and Sqh ‘flow’ towards the centre of the focus (Fig. 4F; Movies 1 and 4).

Interestingly, actin dynamics in LECs involves the formation of distinct regions of contractile activity, depending on LEC behaviour and polarity. Constricting LECs create a single focus in their centre, while migrating LECs generate two alternating foci in their back (Fig. 1B–E). This difference cannot easily be explained by a change in cellular polarity. Instead, it could be due to the different shapes of migrating and constricting cells. In migrating cells, which are elongated along the d-v axis (Fig. S3B,D), one contractile event might not suffice to constrict the whole apicomedial network (Fig. 3F). Constricting cells, however, are rounder (Fig. S3B,D), which might allow radial actin recruitment over the whole apical area (Fig. 4F). Alternatively, the two foci could be a consequence of the cytoskeletal architecture of a migrating cell, as seen in keratinocytes, where myosin localises to two areas in the back of the cell, flanking the nucleus (Wilson et al., 2010).

LECs that create actin foci migrate faster than earlier stage LECs, which do not undergo pulsed contractions (Fig. S2B). However, pulsed contractions are not part of the migratory machinery, as early migrating LECs do not show foci (Fig. 1C; Movie 2) and Rho-CA LECs migrate without pulsatile activity (Fig. 7F; Movie 13). Thus, occurrence of pulsed contractions in migratory LECs appears to be associated with constriction rather than migration.

In contrast to other systems that show pulsed contractions (reviewed in Gorfinkiel and Blanchard, 2011), LECs have a very large apical area, up to 70 µm in diameter. This large area might determine the architecture of the LEC apicomedial network, which consists of persistent actin bundles that connect to the cell cortex, as well as dynamic flows and foci (Figs 1B and 4C–E). In large cells, a robust, persistent apicomedial network might be crucial to maintain apical area and transduce contractile forces. In line with our observations, a persistent and a dynamic pool of actin have recently been described in ectodermal cells during germband extension and in amnioserosa cells during dorsal closure in *Drosophila*, but only the larger amnioserosa cells showed visible actin bundles (Dehapiot et al., 2019).

### The level of contractility affects cytoskeletal dynamics

Altering levels of contractility in LECs showed that pulsed contractions depend on Rok, Myosin phosphatase and Rho1 activity. In this respect, LECs resemble other cells that undergo pulsed contractions (Fischer et al., 2014; Mason et al., 2016; Munjal et al., 2015; Valencia-Expósito et al., 2016; Vasquez et al., 2014). As shown for *Drosophila* germband cells (Munjal et al., 2015) and amnioserosa cells (Fischer et al., 2014), our results indicate that cytoskeletal network dynamics depends on the level of cell contractility (Fig. 8E). Only with intermediate, wild-type levels of contractility did LECs show pulsed contractions. Reducing contractility (Rok-RNAi or MbsN300) interfered with pulsed contractions (Fig. 6A,C), as did increasing contractility (Rho1-CA or Rok-CAT) (Fig. 7E–G). In addition to the amount of activated myosin II, contractility is also determined by the availability of G-actin as well as F-actin nucleators and crosslinkers (Levayer and Lecuit, 2012).

### Increasing contractility also affects cytoskeletal architecture and cell behaviour

As well as interfering with pulsed contractions, increasing contractility in LECs had a more far-reaching impact on cytoskeletal architecture and cell behaviour. (1) Over-expression of Rho1-CA resulted in F-actin disappearing apicomedially and localising mostly to the junctional cortex (Fig. 7F,G). A similar phenotype has been observed during *Drosophila* gastrulation (Mason et al., 2016), suggesting that Rho1 is involved in determining the ratio of apicomedial *versus* cortical contractility (Munjal et al., 2015). (2) While over-expression of Rho1-CA can only activate endogenous Rok, over-expression of Rok-CAT can supply large amounts of activated Rok and thus lead to a stronger and more specific activation of Myosin II. In LECs, this not only resulted in F-actin disappearing apicomedially and localising mostly to the junctional cortex (and thus a loss of pulsed contractions), but also in the formation of cortical actin bundles (Fig. 7D). In cell culture, activation of myosin has also been shown to create actin bundles during stress fibre formation (Chrzanowska-Wodnicka and Burridge, 1996). (3) Increased junctional cortical contractility in both Rho1-CA and Rok-CAT LECs furthermore induced cell blebbing (Fig. 7D,G). Blebbing is driven by strong cortical myosin activation (Paluch et al., 2006). During *Drosophila* dorsal closure, activation of Myosin light chain kinase and Mbs led to cell blebbing (Fischer et al., 2014). (4) In ‘strong’ phenotypes of both Rok-CAT and Rho-CA pupae, LECs do not generate lamellipodia-like protrusions and do not migrate (Fig. 7D,G; Bischoff, 2012). Thus, high levels of activated myosin appear to over-ride protrusive activity and force cells to constrict. A comparable hyper-contractile phenotype has been described in CHO.K1 cells, where over-expressing a phosphomimetic form of myosin light chain leads to a loss of cell spreading and migration (Vicente-Manzanares et al., 2008).

### Rho1 over-expression induces rhythmical cytoskeletal behaviour distinct from pulsed contractions

Increasing wild-type Rho1 levels (*UAS.rho1*) proved particularly interesting. Cells appear to cycle between a state in which actin accumulates at the junctional cortex and cells show blebbing (Fig. 8A″,D), and a state in which the apicomedial network is present but non-pulsatile (Fig. 8A). This cycling phenotype (Fig. 8A; Movie 15) could be explained by the rhythmical activation and de-activation of Rho1 via the endogenous machinery in a context of increased overall contractility of the network. With increasing levels of activated Rho1 (and thus increased levels of activated myosin), LECs appear to shift their ratio of apicomedial versus junctional cortical actin towards cortical. This is reversed when levels of activated Rho1 (and thus levels of activated myosin) decrease. Our results support this hypothesis, as we observed not only the disappearance of apicomedial actin, but also the beginning of junctional cortical blebbing, which suggests an increase in contractility in the junctional cortical network. These observations highlight the importance of specifically coordinating the junctional cortical and the apicomedial actomyosin networks (Garcia De Las Bayonas et al., 2019).

### LECs undergo apical constriction without pulsed contractions

Although pulsed contractions are affected by the manipulation of contractility, we found that most LECs in Rho-RNAi, MbsN300, *UAS.Rho1*, Rho1-CA or Rok-CAT pupae constricted successfully. In all these cases, pulsed contractility of the apicomedial actin network was impaired, but cells still had actin at their junctional cortex (Figs 6A and 7A,D,F,G). This indicates that contractility at the junctional cortex alone can drive apical constriction. Similar observations have been made in amnioserosa cells (Saravanan et al., 2013) and during neural tube formation (Christodoulou and Skourides, 2015). Our observation that boundary LECs constrict without pulsed contractions and with insignificant cell area fluctuation (Fig. 4G,H) further supports the notion that the contractile force needed for constriction can be created by the junctional cortical network, acting like a contractile ring during cell extrusion, as suggested for boundary LEC extrusion (Teng et al., 2017). Another possibility is that the apicomedial network creates non-pulsatile tension that helps drive constriction. For instance, in boundary LECs, diffuse apical Sqh activity can be observed, which might contribute to apical area reduction (Movie 5).

Interestingly, Rok-RNAi LECs labelled with Sqh::GFP showed a phenotype comparable to that of constricting early boundary LECs in controls (Figs 4G and 6A‴; Movies 5 and 8). This raises the possibility that the change from early boundary cell behaviour to the behaviour of cells undergoing pulsed contractions during later stages of development might be due to an increase of contractility in these cells over time.

### The role of pulsed contractions in apical constriction

That LECs can constrict apically without showing pulsed contractions raises important questions about the role of pulsed contractions in constriction, as also raised by others (Blanchard et al., 2010; Xie and Martin, 2015). LECs begin pulsed contractions while still migrating (Fig. 1C), at a time when LEC shape changes, and thus tissue remodelling intensifies (Fig. S3B). Also, for most of morphogenesis, LECs undergo pulsed contractions without apically constricting, merely fluctuating their cell area rhythmically and changing shape (Fig. 2A; Fig. S3B–D). This suggests that pulsed contractions do not drive apical constriction per se. Instead, they might play other roles, such as helping to maintain cell shape and to withstand pushing and pulling forces created by neighbouring cells during morphogenesis. Ultimately, this could help to maintain tissue integrity during morphogenesis (Coravos et al., 2017). Alternatively, pulsed contractions could cooperate with junctional cortical contractility to create sufficient forces to drive apical constriction more effectively; LEC constriction that is accompanied by pulsed contractions is faster than constriction of boundary LECs without actin foci (Fig. 4G). To gain further insights into the role of pulsed contractions in LECs, future studies need to consider the interactions between neighbouring LECs to assess the impact of external forces on the behaviour of the apicomedial actomyosin network.

### Concluding remarks

Our study provides insights into the complexity of the cytoskeleton of a cell during morphogenesis (Fig. 5), as well as the importance of the level of cell contractility for the regulation of pulsed contractions, cellular architecture and, consequently, cell behaviour (Fig. 8E). It also highlights the complex interplay between the junctional cortical and the apicomedial network in changing cell shape and cell area. For LEC migration and the subsequent behavioural transition to constriction, a polarised cytoskeletal network and intermediate contractility levels seem to be crucial. What regulates the transition from migration to constriction is unknown. As LECs transit from PCP to RCP while they change behaviour, studying signals that regulate this change in polarity will be crucial in future studies.

## MATERIALS AND METHODS

### Fly stocks

FlyBase (Gramates et al., 2016) entries of the used transgenes are as follows:*hh.Gal4*: *Scer/Gal4^hh-Gal4^*, *UAS.gma-GFP*:*Moe^Scer\UAS.T:Avic\GFP-S65T^*, *UAS.LifeAct-Ruby*: *Scer\ABP140^Scer\UAS.T:Disc\RFP-Ruby^*, *sqh::GFP*: *sqh^RLC.T:Avic\GFP-S65T^*, *sqh^−/−^*: *sqh^Ax3^*, *UAS.MbsN300*: *Mbs^N300.Scer\UAS^*, *UAS.rok-RNAi*: *Rok^KK107802^* (VDRC 104675; Dietzl et al., 2007), *UAS.rok-*CAT: *Rok^CAT.Scer\UAS^*, *UAS.rho1-CA*: *Rho1^V14.Scer\UAS^*, *UAS.rho1: Rho1^UAS.cMa^*, *tub-FRT-CD2-FRT-Gal4*: *Rnor\CD2^A902^*, *UAS.mCD8-GFP*: *Mmus/Cd8a^Scer\UAS.T:Avic/GFP^*, *UAS.FLP*: *FLP1^Scer\UAS.cDa^*, *tub.Gal80ts*: *Scer\Gal80^ts.αTub84B^*, hs.FLP:*FLP1^hs.PS^*, Rok::GFP: *sqh.GFP-Rok*.

For the sqh::GFP experiments, we used *sqh[Ax3]; sqh::GFP; sqh::GFP* flies. Flies with one copy of sqh::GFP (*sqh[Ax3]/w; +/+; sqh::GFP/+*) showed similar labelling (Fig. S4D). The Rok-RNAi line has been used before (Rousso et al., 2015).

### Expression of transgenes in LECs

To express *UAS*-transgenes in the posterior compartment, *hh.Gal4* was used. If transgenes were lethal when expressed throughout development, we used *tub.Gal80ts* to repress expression until 30–50 h before imaging (*UAS.rok-RNAi*, *UAS.MbsN300*, *UAS.rok-CAT*, *UAS.rho1-CA*, *UAS.rho1*). Repression was released by shifting flies to the restrictive temperature (29°C) until imaging. The behaviour of the cytoskeleton was not altered by Gal80ts presence; foci were localised in the back of migrating LECs and in the centre of constricting LECs (Fig. S6B). For the *UAS.rok-CAT* experiments, we used different temperatures to release transgene repression. At the restrictive temperature, 35.3% of pupae showed a weak phenotype (*n*=17). At 25°C, where repression of Gal4 by Gal80ts is leaky, 100% of pupae showed a weak phenotype (*n*=5). All *UAS.rho1* pupae were grown at 25°C.

### Microscopy

For 4D microscopy, pupae were staged according to Bainbridge and Bownes (1981). Pupae were dissected and filmed as described in Seijo-Barandiarán et al. (2015). In all images and movies, anterior is to the left. All flies developed into pharate adults and many hatched. *z*-stacks with a step size of 0.5 to 2.5 µm were recorded every 2 to 150 s, depending on the experiment. Imaging was done with a Leica SP8 confocal microscope at 25±1°C with HCPL APO CS2 20×/0.75 IMM, HCPL APO CS2 40×/1.30 Oil or HCPL APO CS2 63×/1.40 Oil objectives, respectively. Image size was 512×512 pixels or 1024×1024 pixels. All images and movies shown are maximum intensity projections of *z*-stacks, if not mentioned otherwise. Figures and movies were made using Adobe Illustrator, Adobe Photoshop, Adobe Media Encoder (Adobe Inc., San Jose, CA), ImageJ (NIH, Bethesda, MD), Fiji (Schindelin et al., 2012) and Leica LAS AF Lite (Leica Microsystems, Mannheim, Germany).

### Quantitative analysis of 4D movies

All LECs showed pulsed contractions and migratory and constrictive behaviour. We focused our analysis on the dorsal side of segment A2, in a region at the back of the P compartment, three LEC rows away from the histoblasts at 18 h APF (Fig. S1A′). This allowed comparability of individual cells, as cell shape, cell size and timing of delamination vary in different regions of the tissue.

#### Tracking of actin foci

Actin foci were tracked manually every 30 s using the software SIMI Biocell (SIMI, Unterschleissheim, Germany) (Schnabel et al., 1997). We defined an actin focus as the coalescence of high GMA-GFP or Sqh::GFP fluorescence, with fluorescence moving to the location of the focus from two or more regions of the cell, followed by disassembly, with actin flowing in various directions away from the focus. Assembly, focus and disassembly had to be visible in at least three consecutive frames. We defined actin flows as high GMA-GFP or Sqh::GFP fluorescence that moved around in the cell without or before creating an actin focus. Movies for tracking were recorded over night with a time interval of 30 s and a *z*-interval of 1 µm.

#### Calculating the relative position of actin foci

In addition to actin foci, the most anterior, posterior, dorsal and ventral coordinates of LECs were also tracked manually. These coordinates were used to calculate the relative position (RP) of foci in the cells along the a-p and the d-v axis (Eqns 1 and 2):(1)
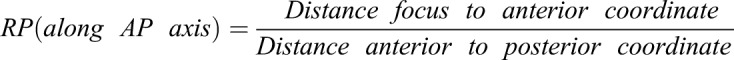
(2)
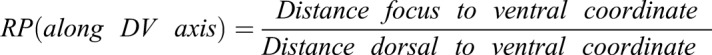


#### Calculating periodicity of actin foci

Actin foci were tracked and the time difference between two consecutive foci was calculated (from full contraction to full contraction).

#### Calculating of cell shape coefficient

To obtain a single value that describes the shape of the cell, the cell shape coefficient was calculated using the most anterior, posterior, dorsal and ventral coordinates (Eqn 3):(3)
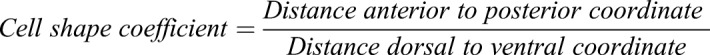
This coefficient assumes values close to 1 when the cell is round, and close to 0 when the cell is thin and long along the d-v axis.

#### Measuring cell area over time

Cell area was tracked manually using ImageJ. The polygon selection tool was used to draw the cell area, using as many vertices as needed to have an accurate outline. From one frame to the next, this selection was adjusted if cell area changed over time. To estimate the error associated with this technique, we tracked a cell for three frames and repeated the tracking 22 times. We found that the average error between repetitions was 0.37±0.07% (mean±s.d). For seven wild-type LECs, cell area was measured every frame (30 s) for the entire length of the recording, covering all four behavioural phases (Figs 2 and 3; Fig. S3). For the analysis of mutant LECs, we measured a 30 min window of migratory and constrictive behaviour [beginning 25 min before lamellipodium disappearance (migration phase) and 75 min after lamellipodium disappearance (constriction phase)] (Figs 6D and 7C; Fig. S6A). This allowed higher throughput. For the tracking of Rho-CA and Rok-CAT LECs we considered cell area excluding blebs.

#### Calculating reduction in cell area per contraction

The amplitude of the cell area fluctuations was calculated by manually identifying the crests and the troughs for each fluctuation cycle and calculating the difference (scheme in Fig. 2B). Then the percentage by which the cell area is reduced in each fluctuation was calculated (Eqn 4):(4)
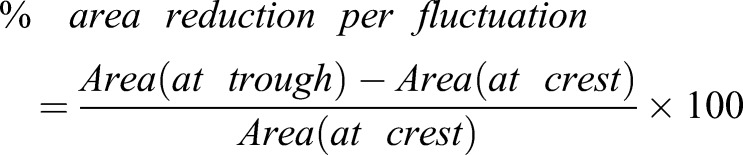
Differences in cell area reduction per contraction between the different phases of LEC behaviour are not due to differences in cell size, as similar results are obtained when considering percentage area reduction and area reduction in µm^2^ (compare Fig. 2C with Fig. S3A; and Fig. 6B with Fig. S6A).

#### Assessing the number of actin foci per area fluctuation and the relationship between foci and area fluctuation

The number of actin foci per area fluctuation was calculated by manually counting the presence of foci tracked between the crests of each area fluctuation. To assess the relationship between actin foci and area fluctuation, we measured the time interval between a focus and the nearest trough of the area fluctuation by subtracting the time point at which the actin focus accumulates and the time point of the nearest trough for each tracked focus (scheme in Fig. 3C).

#### Measuring junctional fluorescent intensity of Sqh::GFP

Junctional fluorescence intensity was measured in ImageJ. For all quantifications, sum-intensity *z*-projections of two to four *z*-slices were used. For each analysed cell, a line of 8–10 µm was drawn manually on top of an a-p and a d-v interface to measure the fluorescence intensities of the junctions. Measurements were done at two time-points: at the onset of migration, when cells clearly show a lamellipodium and start to move, and during apical constriction. All cells from a chosen image that were positioned in the P compartment and did not lie at the compartment or segment boundary and which were not obstructed by folds in the pupal cuticle were analysed. We excluded cells at the segment and compartment boundaries as they had higher sqh::GFP intensities due to the formation of actin cables at those boundaries. For comparing junctional intensity values between a-p and d-v interfaces, the intensity measurement from each junction was normalized to the mean of all measured junctions of the respective pupae as well as the respective behaviour (migration or constriction). This normalization was done to account for differences in fluorescence intensity between different pupae.

#### Co-localisation analysis of LifeAct-Ruby, Sqh::GFP and Rok::GFP

Using ImageJ, a region of 20 µm^2^ was drawn in the cell centre. Relative fluorescence intensities in this region were calculated for each channel using the plot profile function (Fig. 4A,B). This function creates an average *y*-projection of the selected region before measuring intensities along the resulting line.

#### Sector analysis of fluorescence

Using ImageJ, fluorescence intensities were measured over time for nine sectors (8.2 µm^2^ each) covering the cytoplasm of a LEC, using the plot profile function (Fig. 4F). Data were used to create a plot in Excel (Microsoft Office, Microsoft Corporation).

#### Cell size determination

As cells change shape dynamically and no two cells are in exactly the same stage of development at the same time, we needed to make sure that cell sizes could still be compared. For this, we measured the largest P compartment cell during migration and/or constriction using the polygon tool of ImageJ (Fig. 6B).

#### Kymographs

Kymographs were used to present cytoskeletal dynamics in a single image (Figs 6C, 7B,E–G and 8A‴,D). Kymographs were created from a rectangular region of interest (ROI), using the ImageJ tool ‘Reslice’, followed by a maximum *z*-projection of the obtained stack. This operation leads to vertical pixel rows, each of which depicts a *y*-projection of the ROI for each frame of the analysed movie (scheme in Fig. 6C). These rows were stacked on top of each other to illustrate the relative fluorescence intensity change in the ROI over time.

#### Trajectory plots

Trajectory plots (Fig. S5) were created using SIMI Biocell.

#### Quantification of abdominal closure timing and abdominal closure defects

For quantifying abdominal closure defects and assessing closure timing, *UAS*-transgenes were expressed in all LECs, as previously described (Bischoff, 2012). In short, FLP-out clones (Struhl and Basler, 1993) were induced by heat shocking third instar larvae for 15 min at 37°C. This led to transgene expression in all LECs but rarely in the histoblasts, as LECs are polyploid (Ninov et al., 2007). Expression of *UAS.FLP* increased recombination events in the polyploid LECs, thus increasing expression. After heat shock, flies were kept at 25°C for 1–2 days before imaging.

To quantify how long it takes for LECs to complete abdominal closure (Fig. 6F′), we measured the time from the end of posterior migration (i.e. disappearance of posteriorly directed lamellipodia and start of histoblast nest expansion) to delamination of the last LEC.

#### Quantification of the duration of states of apicomedial and junctional cortical actin localisation in *UAS.Rho1* pupae

To quantify cycling of LECs between the two phases (Fig. 8A‴,B), fluorescence intensity was measured in a 23 µm^2^ area in a central apical region of the cells over time using ImageJ (see plot in Fig. 8A‴). The resulting data were used to manually identify the two phases (‘high’ and ‘low’ fluorescence), as well as the transitions between the two phases, which were defined by a sudden drop/increase in fluorescence intensity with intensity values that lay outside the range of intensities found during the ‘high’ or ‘low’ phases.

#### Quantification of migration speed

To calculate migration speed of cells in phases 1 and 2 (Fig. S2B), cells were tracked using SIMI Biocell (*n*=6 LECs in individual pupae). Migration speed was calculated for each 6 min interval throughout phases 1 and 2.

### Animal models and statistical analysis

All used *Drosophila melanogaster* stocks are described above. Staged pupae were used for the experiments. The sex of the experimental flies was not determined.

Statistical analyses were carried out in R (R Core Team, 2016) and Prism 8 (GraphPad Software, San Diego, CA), assuming a significance level of 0.05. To establish the number of pupae required for detecting differences in the localisation of foci between the different phases (Fig. 1D), we performed a power test using power.t.test (R Stats Package, version 3.6.1). For all other experiments, sample sizes were determined by the available material that could be processed during each experiment. The experiments were not randomised and the investigators were not blinded during analyses. No analysable samples were excluded; cells in some movies were not analysable, due to drift of the recording or a fold in the above pupal cuticle obscuring the cells. To determine the appropriate statistical tests, the data sets were tested for normal distribution and for homogeneity of variance.

For normally distributed data, two-sample Student's *t*-tests were used to compare the means of two groups. For more than two groups, one-way analyses of variance (ANOVA) tests were conducted. For non-parametric data of two groups, Mann–Whitney *U*-tests were used. For non-parametric data of more than two groups, Kruskal–Wallis H tests were used, followed by a pairwise Wilcoxon–Mann–Whitney test, χ^2^ test or Dunn’s test for multiple comparisons. Medians were used for the non-parametric data, as they are better measures of the central tendency of the data for skewed distributions. In order to calculate the standard errors and confidence intervals for the medians (in Figs 2C,D, 3E, 4I and 6D; Figs S3A and S6), a bootstrap method was applied (Efron, 1979), using a plug-in in R. The number of replications chosen to obtain an accurate estimate was 1000. If not mentioned in the main text, *n* numbers are given in the figure legends and details about the statistical tests are listed in Table S1. *n* numbers of confocal micrograph data are given in Table S2. If not mentioned otherwise, box plots were plotted with minimum, first quartile (Q1), median, third quartile (Q3) and maximum.

## Supplementary Material

Supplementary information
