# Supplementary material for: Coordination of cytoskeletal dynamics and cell behaviour during Drosophila abdominal morphogenesis
Source: J Cell Sci. 2020 Mar 30;133(6):jcs235325. doi: 10.1242/jcs.235325 (PMC7132776; doi:10.1242/jcs.235325)
Supplement: Supplementary information [file joces-133-235325-s1.pdf]

Supplementary Material

Supplementary Figures

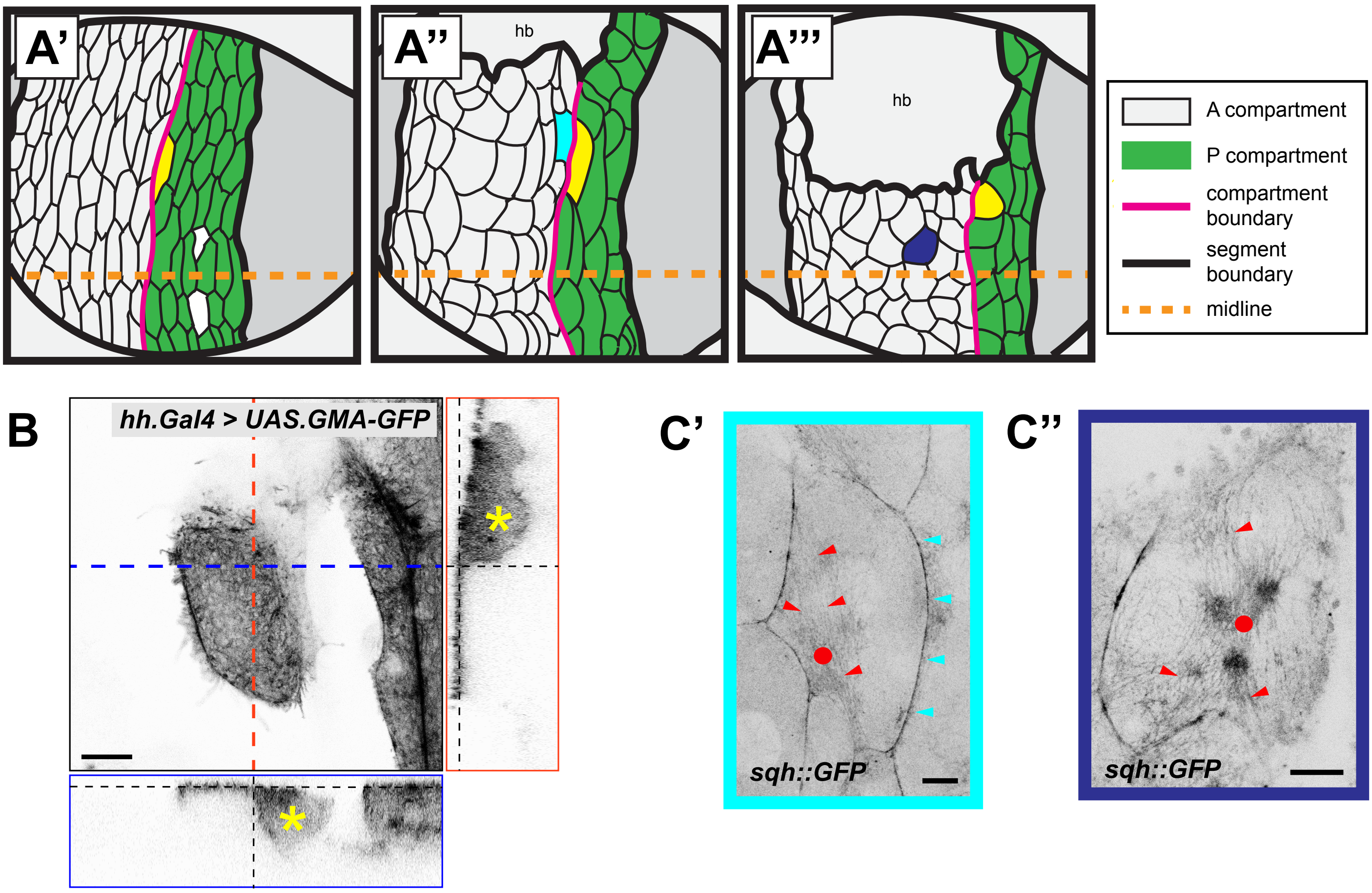

Supplementary Figure S1

Journal of Cell Science • Supplementary information

Figure S1. The apicomedial cytoskeletal network of LECs undergoes pulsed contractions. (**A**) Abdominal epithelial morphogenesis. Schemes of abdominal epithelium (segment A2) before the start of morphogenesis (A'), during migration (A'') and during constriction (A'''). Note that LECs create crescent-shaped lamellipodia when they migrate collectively in posterior direction (A'') (Bischoff, 2012). The integrity of the epithelium is never disrupted and LECs can only migrate, if neighbours make space by either migrating, getting narrower or undergoing apoptosis (Bischoff, 2012). Hence, cell behaviour varies across the epithelium, with cells at the segment boundaries getting thinner and cells in the centre of the segment increasing their cell area (Bischoff, 2012). We focussed our analysis on LECs of the P compartment, two rows lateral to the dorsal midline (yellow cell). hb, histoblasts. Cells coloured cyan and blue, respectively, shown in (C). (**B**) LECs mainly consist of a large apical area with an apical actin network; F-actin labelled with GMA-GFP. The basal cell body surrounds the nucleus (asterisk). Red, *z*-section along d-v axis. Blue, *z*-section along a-p axis. (**C**) Cells in the A compartment also show pulsed contractions, both during migration (e.g. cyan cell in A''; cyan box) and constriction (e.g. blue cell in A'''; blue box). LECs labelled with Sqh::GFP. Red dots, assembling actin foci; red arrowheads, actin bundles; cyan arrowheads, lamellipodium; Scale bars, 10 µm. In all panels, anterior is to the left.

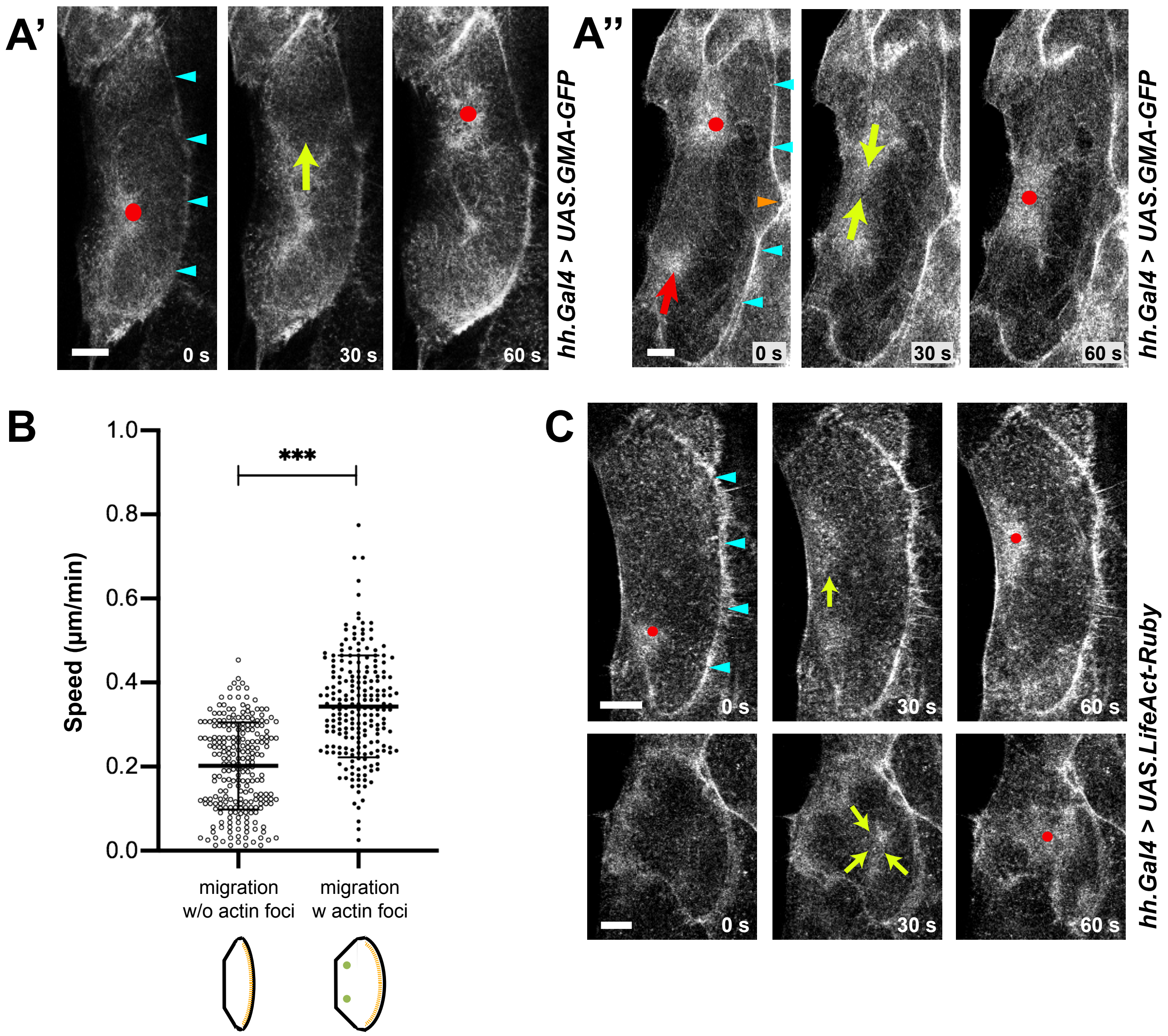

Supplementary Figure S2

Journal of Cell Science • Supplementary information

Figure S2. The behaviour of the LECs during migration and constriction. (**A**) Actin flow patterns change when LECs transit from migration to constriction. GMA-GFP labels F-actin. Red dot, actin focus; yellow arrow, direction of actin flows; cyan arrowheads, lamellipodium. (A') Typical pattern of actin foci and flows during migration – foci alternate between two positions and the flows move between these positions. (A'') During the transition from migration to constriction, disruption of lamellipodium shape (orange arrowhead) indicates the beginning of its disappearance. Actin flow patterns change: From the locations of the assembling (red dot) and disassembling (red arrow) foci, flows move towards the cell centre where the subsequent focus then forms. (**B**) Comparing the speed of migration of LECs without actin foci (phase 1) and with actin foci (phase 2). LECs undergoing pulsed contractions migrate significantly faster. n (phase1)=237, n (phase2)=212; violin plot, mean±s.d.; P***<0.001. (**C**) Pulsed contractions and flows labelled with LifeAct-Ruby. Top, migration; bottom, constriction. Red dot, actin focus; yellow arrow, direction of actin flows; cyan arrowheads, lamellipodium.

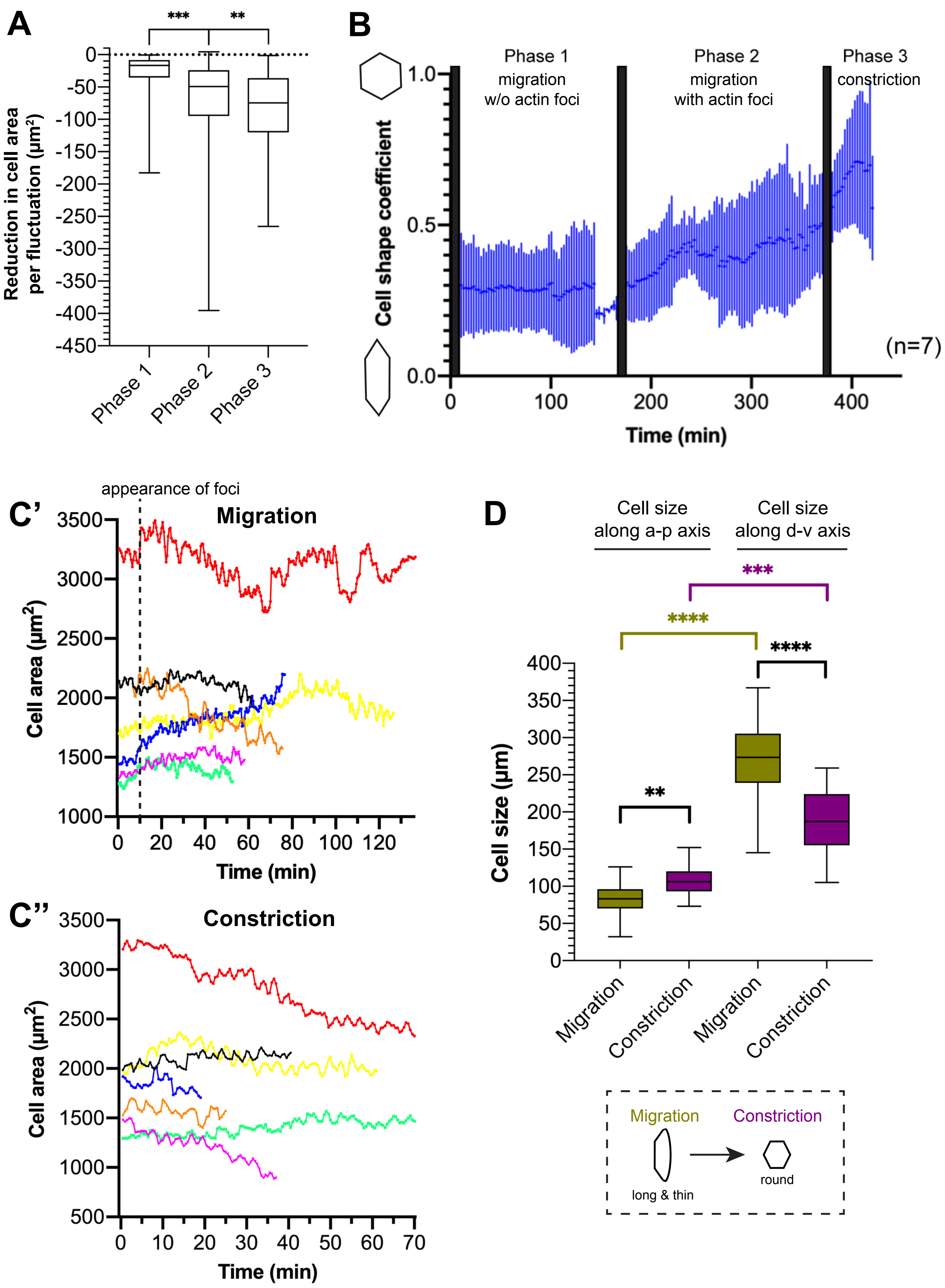

Supplementary Figure S3

Journal of Cell Science • Supplementary information

Figure S3. Change in LEC shape over time. (**A**) Boxplot comparing the reduction in cell area per pulsed contraction in phases 1, 2 and 3. *n* (phase1)=379 fluctuations in seven LECs from individual pupae, *n* (phase2)=223 fluctuations in seven LECs from individual pupae; *n* (phase3)=149 fluctuations in seven LECs from individual pupae; ****P*<0.001, ***P*<0.01. **(B**) Change in cell shape over time. Cell shape coefficient plotted over time (mean±s.d.; n=7 LECs in individual pupae). The beginning of the three phases is adjusted, so each phase begins at the same time for all cells (black bars). Two of the LECs have a longer phase 1, so the short errors bars at the end are due to these two cells. (**C**) Cell area change over time for seven individual LECs. (C') End of early migration phase and late migration phase until disappearance of lamellipodium shown. The length of phase 2 varies between cells. (C'') Around the first hour of the early constriction phase shown. Note that during both migration and early constriction, cell area is not reduced notably, although cells show apical area fluctuations. During constriction, some cells start reducing their apical area earlier than others, therefore, the early constriction phase is shorter. (**D**) Cell shape change during the transition from migration to constriction. Box plot showing the a-p and d-v length of LECs during migration and constriction. Cells are long and thin during migration and round during constriction. Most of the cell shape change is due to a shortening of the cells' d-v axis. *n* (migration)=278 time points in seven LECs in individual pupae, *n* (constriction)=123 time points in seven LECs in individual pupae; *****P*<0.0001, ****P*=0.0005. Scale bars, 10 μm. Anterior, left; dorsal, top.

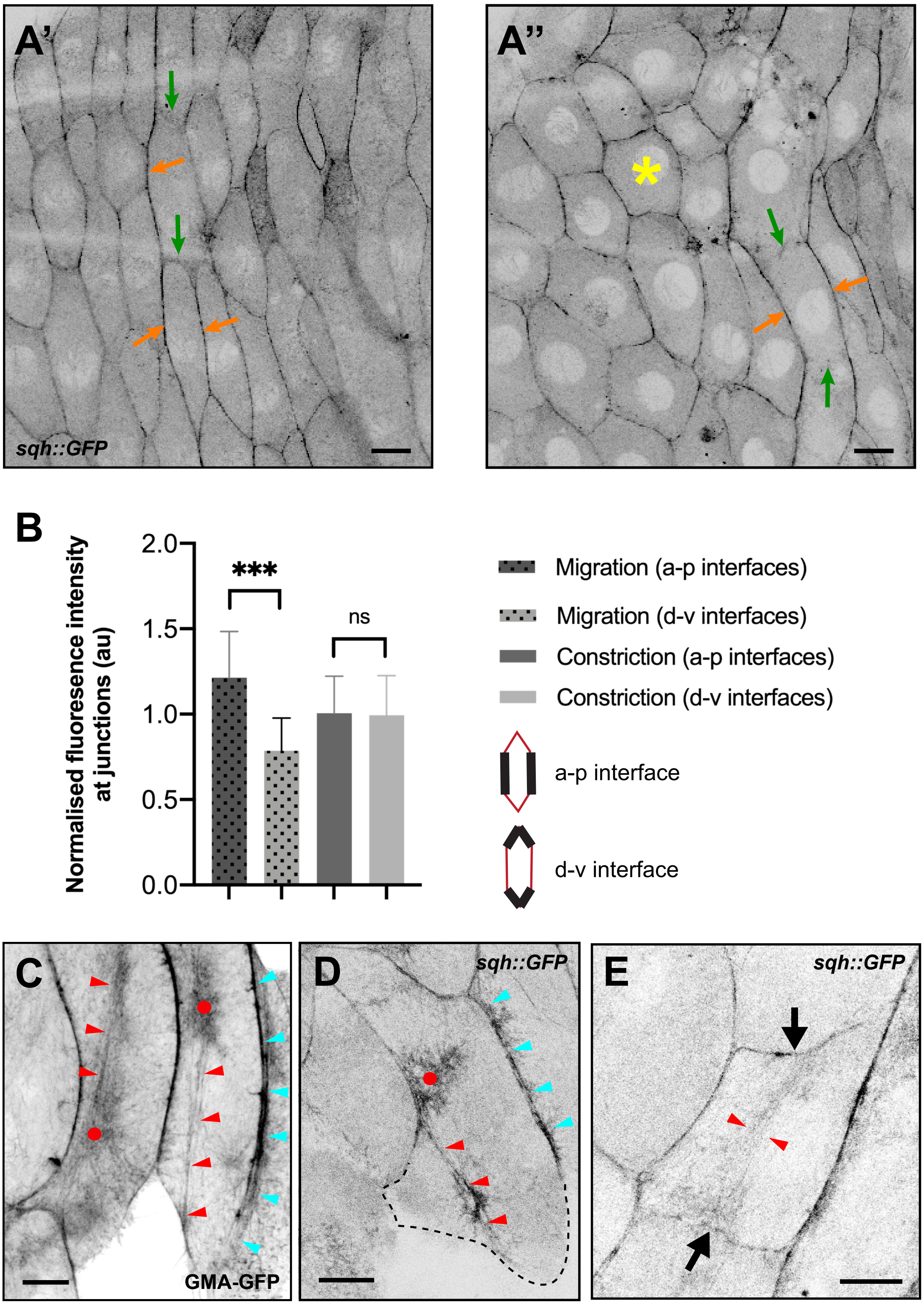

Supplementary Figure S4

Journal of Cell Science • Supplementary information

Figure S4. Sqh::GFP localisation during morphogenesis. (**A**) Overview of abdominal segment A2. (A') At the beginning of morphogenesis, LECs preferentially localise Sqh::GFP at their a-p cell-cell interfaces (orange arrows), rather than their d-v interfaces (green arrows). (A'') During constriction (phase 3), cells that have not yet begun to constrict still localise Sqh::GFP preferentially at the a-p cell-cell interfaces (orange arrows), rather than the d-v interfaces (green arrows). Cells that are constricting have a more even Sqh::GFP localisation at all interfaces (asterisk). **(B)** Quantification of junctional Sqh::GFP fluorescence intensity. Migrating LECs show significantly higher Sqh::GFP fluorescence in their a-p junctions compared to their d-v interfaces. In constricting LECs, this difference is lost. *n* (migration)=58 junctions in 29 cells in seven pupae, *n* (constriction)=52 junctions in 26 cells in seven pupae; bar plot with s.d.; ****P*<0.0001. **(C)** Migrating GMA-GFP LECs showing actin foci (red dots) and actin bundles in the back (red arrowheads). Cyan arrowheads, lamellipodium. (**D**) Migrating LEC with two copies of Sqh::GFP shows focus (red dot) and actin bundles in the back (red arrowhead), as well as a lamellipodium at the front (cyan arrowheads). Dotted line indicates cell outline. (**E**) Constricting LECs occasionally show actin bundles that are oriented along the d-v axis (red arrowheads). These bundles pull at the dorsal and ventral membranes (black arrows). Scale bars, 10 µm. Anterior, left; dorsal, top.

Journal of Cell Science • Supplementary information

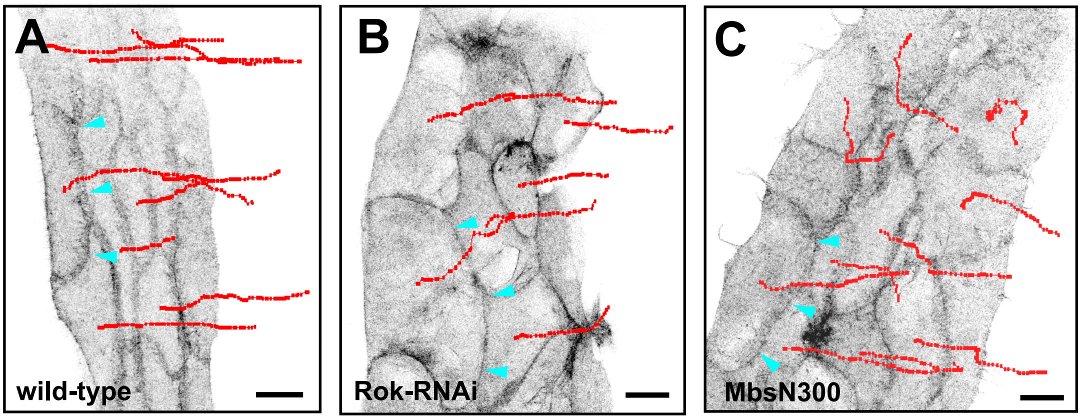

Supplementary Figure S5

Figure S5. Rok-RNAi and MbsN300 LECs migrate normally in posterior direction. GMA-GFP labels F-actin. Tracks of cells shown in red; cyan arrowheads, lamellipodia. (**A**) GMA-GFP control (*n*=7 pupae). (**B**) Rok-RNAi (*n*=4 pupae). (**C**) MbsN300 overexpression (*n*=4 pupae). Scale bars, 20 μm. Anterior, left; dorsal, top.

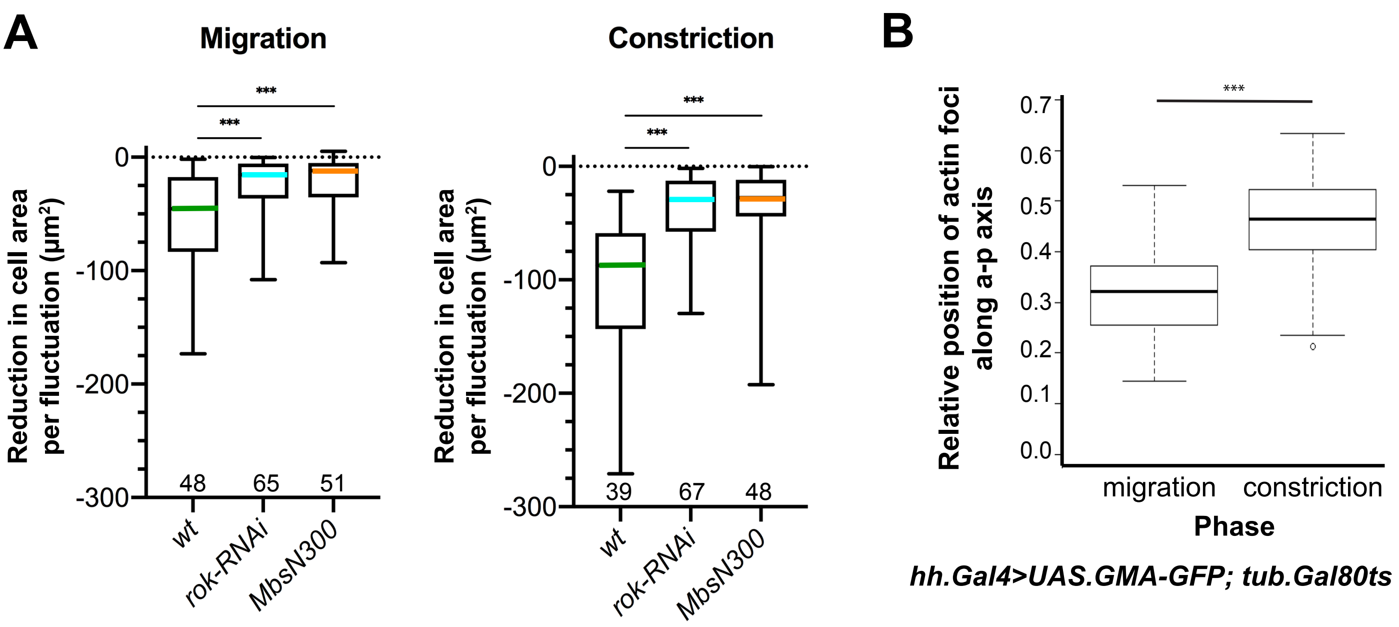

Supplementary Figure S6

Figure S6. Additional quantitative analyses. (**A**) Boxplot comparing reduction in cell area per pulsed contraction in wild-type, Rok-RNAi and MbsN300 overexpression during migration and constriction (*n* (foci) given along *x*-axis). Migration: *n* (*wt*)=48 fluctuations in four LECs in individual pupae, *n* (*rok-RNAi*)=65 fluctuations in five LECs in individual pupae, *n* (*MbsN300*)=51 fluctuations in four LECs in individual pupae. Constriction: *n* (*wt*)=39 fluctuations in four LECs in individual pupae, *n* (*rok-RNAi*)=67 fluctuations in five LECs in individual pupae, *n* (*MbsN300*)=48 fluctuations in three LECs in individual pupae. ****P*<0.001. (**B**) Expression of Gal80ts does not alter positioning of actin foci. Boxplot showing relative position of actin foci along the a-p axis in LECs that express *UAS.GMA-GFP* in a *tub.Gal80ts* background. In migrating cells, actin foci are found in the back, while in constricting cells, actin foci are located in the centre. The period between pulses is comparable to wild-type (180 ±0.2s; median±s.e.m.; *n*=200 pulses in five individual pupae). *n* (migration)=112 foci in five LECs in individual pupae, *n* (constriction)=109 foci in five LECs in individual pupae; ****P*<0.001.

Table S1. Details of statistical tests used.[a]

Figure	Statistical test with details and *P*-value
Fig. 1D	*GMA-GFP:* Anova, F(1,399)=449.8, ****P*<0.001<br>*LifeAct-Ruby:* Anova, F(1,319)=447.7, ****P*<0.001
Fig. 1F’	Anova, F(1,9)=27.99, ****P*<0.001
Fig. 2C	Kruskal Wallis Rank Sum test (χ^2=94.61, d.f.=2), post-hoc Wilcoxon-Rank sum test, P-adjustment method Bonferroni:<br>*phase 1 vs. phase 2:* ****P*<0.001<br>*phase 1 vs. phase 3:* ****P*<0.001<br>*phase 2 vs. phase 3:* ****P*<0.001
Fig. 2D	Kruskal Wallis Rank Sum test (χ^2=23.70, d.f.=1), ****P*<0.001
Fig. 3E	*Short fluctuations:*<br>migration *vs*. constriction: Kruskal Wallis Rank Sum test (χ^2=0.50, d.f.=1), *P*=0.48<br>*Longer fluctuations:*<br>migration *vs.* constriction:<br>Kruskal Wallis Rank Sum test (χ^2=0.5, d.f.=1), ****P*<0.001<br>*Short migration vs. long migration:*<br>Kruskal Wallis Rank Sum test (χ^2=35.27, d.f.=1), ****P*<0.001<br>*Short constriction vs. long constriction:*<br>Kruskal Wallis Rank Sum test (χ^2=29.14, d.f.=1), ****P*<0.001
Fig. 4I	Mann Whitney test U=112, Sum of ranks=3359, 2527, two-tailed, ****P*<0.001
Fig. 6B	*Migration:*<br>Anova, F(24,2)=15.46, pairwise comparisons using t tests with pooled s.d.: *wt vs rok-RNAi*: ****P*<0.001, *wt vs MbsN300*: ***P*=0.02; *MbsN300 vs rok-RNAi*: *P*=0.34<br>*Constriction:*<br>Anova, F(24,2)=39.45, pairwise comparisons using t tests with pooled s.d.: *wt vs rok-RNAi*: ****P*<0.001, *wt vs MbsN300*: ***P*<0.01, *MbsN300 vs rok-RNAi*: ***P*<0.01<br>*Late constriction:*<br>Anova, F(25,2)=51.91, pairwise comparisons using t tests with pooled s.d.: *wt vs rok-RNAi*: ****P*<0.001, *wt vs MbsN300*: ***P*<0.01, *MbsN300 vs rok-RNAi*: *P*=0.11
Fig. 6D	*Migration:*<br>Kruskal Wallis Rank Sum test, χ^2=43.37, d.f.=2, post-hoc Wilcoxon-Rank sum test, P-adjustment method: Bonferroni:<br>*wt vs. Rok-RNAi*: ****P*<0.001, wt *vs.* MbsN300: ****P*<0.001, *rock-RNAi vs. MbsN300: P*=1<br>*Constriction:*<br>Kruskal Wallis Rank Sum test, χ^2=70.89, d.f.=2, post-hoc Wilcoxon-Rank sum test, P-adjustment method: Bonferroni:<br>*wt vs. Rok-RNAi*: ****P*<0.001, wt *vs.* MbsN300: ****P*<0.001, *rock-RNAi vs. MbsN300: P*=0.56
Fig. 6F	Kruskal Wallis test, H(3)=8.859, *P*<0.005; post-hoc test Dunn’s test for multiple comparisons:<br>*wt vs. rok-RNAi*: z=2.534, **P*<0.05, *wt vs. MbsN300*: z=2.793, **P*<0.05; *wt vs. MbsN300*: z=0.3866, n.s. *P*>0.999

Figure	Statistical test with details and p-value
Fig. 8B	Mann-Whitney test, U=3685, sum of ranks=7426, 7453, two-tailed, *P*=0.968, nine outliers excluded from plot (Left: 52.5, 67, 72.75; Right: 67, 75.75, 110.25, 156, 195.75, 209.25)
Fig. S2B	Mann-Whitney test, U=9741; two-tailed; sum of ranks=37944, 63082, *P****<0.001.
Fig. S3A	Kruskal Wallis Rank Sum test (χ^2=211.05, d.f.=2), post-hoc Wilcoxon-Rank sum test, P-adjustment method Bonferroni:<br>*phase 1 vs. phase 2:* ****P*<0.001<br>*phase 1 vs. phase 3:* ****P*<0.001<br>*phase 2 vs. phase 3:* ***P*<0.01
Fig. S3D	Kruskal Wallis test, H(6)=1118, *P*<0.0001, post-hoc test Dunn's test for multiple comparisons:<br>*migration a-p vs. constriction a-p:* z=3.609, ***P*=0.0046<br>*migration a-p vs. migration d-v:* z=16.62, *****P*<0.0001<br>*migration d-v vs. constriction d-v:* z=4.516, *****P*<0.0001<br>*constriction a-p vs. constriction d-v:* z=4.155, ****P*=0.0005
Fig. S4B	Kruskal Wallis test, H(4)=51.07, *P*<0.0001; post-hoc test Dunn's test for multiple comparisons:<br>*migration a-p vs. migration d-v:* z=6.714, ****P*<0.001<br>*constriction a-p vs. constriction d-v:* z=0.1048, *p*>0.999
Fig. S6A	*Migration:*<br>Kruskal Wallis Rank Sum test, χ^2=24.76, d.f.=2, post-hoc Wilcoxon-Rank sum test, P-adjustment method: Bonferroni:<br>*wt vs. Rok-RNAi*: ****P*<0.001, wt *vs. MbsN300*: ****P*<0.001, *rok-RNAi vs. MbsN300*: *P*=1<br>*Constriction:*<br>Kruskal Wallis Rank Sum test, χ^2=47.78, d.f.=2, post-hoc Wilcoxon-Rank sum test, P-adjustment method: Bonferroni:<br>*wt vs. Rok-RNAi*: ****P*<0.001, wt *vs.* MbsN300: ****P*<0.001, *rok-RNAi vs. MbsN300*: *P*=1)
Fig. S6B	Anova, F(1,219)=168.2, ****P*<0.001

[a] Sample sizes in figure legends.

Table S2. *n*-numbers of confocal micrograph data.

Experiment	*n*-number	Figure
hh.Gal4 > UAS.GMA-GFP		
- high magnification	8[a]	1B; 6A,E; S1B; S4C
hh.Gal4 > UAS.LifeAct-Ruby	7 [a]	1D; S2C
sqh::GFP		
- tissue overview	6[b]	S4A
- high magnification	17[a]	4C-E; S4E
- Sqh::GFP *(one copy)*	20[b]	S4D
sqh[Ax3]; sqh::GFP/UAS.LifeAct-Ruby; sqh::GFP/+	14[a]	4A
rok::GFP/UAS.LifeAct-Ruby	20[a]	4B
hh.Gal4 > UAS.rok-RNAi		
- high magnification	11[a]	6A’’
hh.Gal4 > UAS.MbsN300		
- high magnification	13[a]	6A’’’’

[a] single LEC in individual pupa
[b] pupae

Journal of Cell Science • Supplementary information

Supplementary Movie

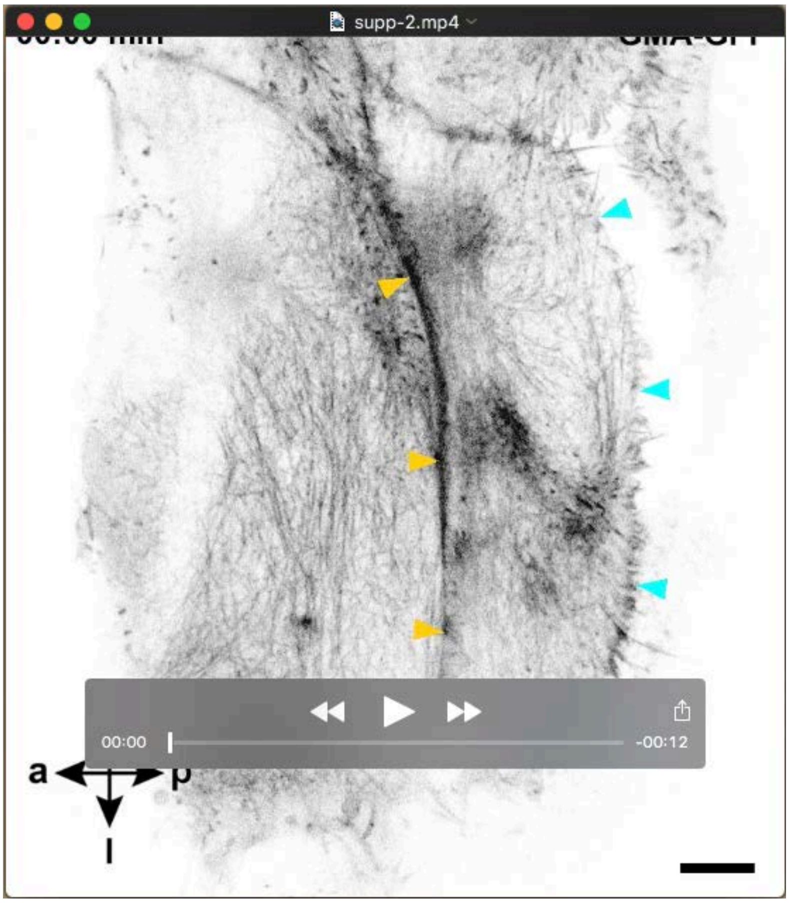

Movie 1. LECs undergo pulsed contractions that correlate with their behaviour. Confocal micrographs of two LECs labelled with GMA-GFP undergoing pulsed contractions. Right cell is migrating, showing a lamellipodium (cyan arrowheads), and contracting at the same time. Left cell has lost its lamellipodium, has stopped migrating and is only constricting – orange arrowheads indicate its posterior cell interface that is more flexible than the posterior interface of its neighbour, where the lamellipodium appears to stabilise the cell-cell interface through its protrusive activity. The migrating cell shows two alternating actin foci, the constricting cell shows one focus (red dots indicate foci). Red arrow, actin bundles in the back of the migrating cell. Scale bar, 10 µm. a, anterior; p, posterior; m, medial (corresponds to dorsal); l, lateral (corresponds to ventral).

Journal of Cell Science • Supplementary information

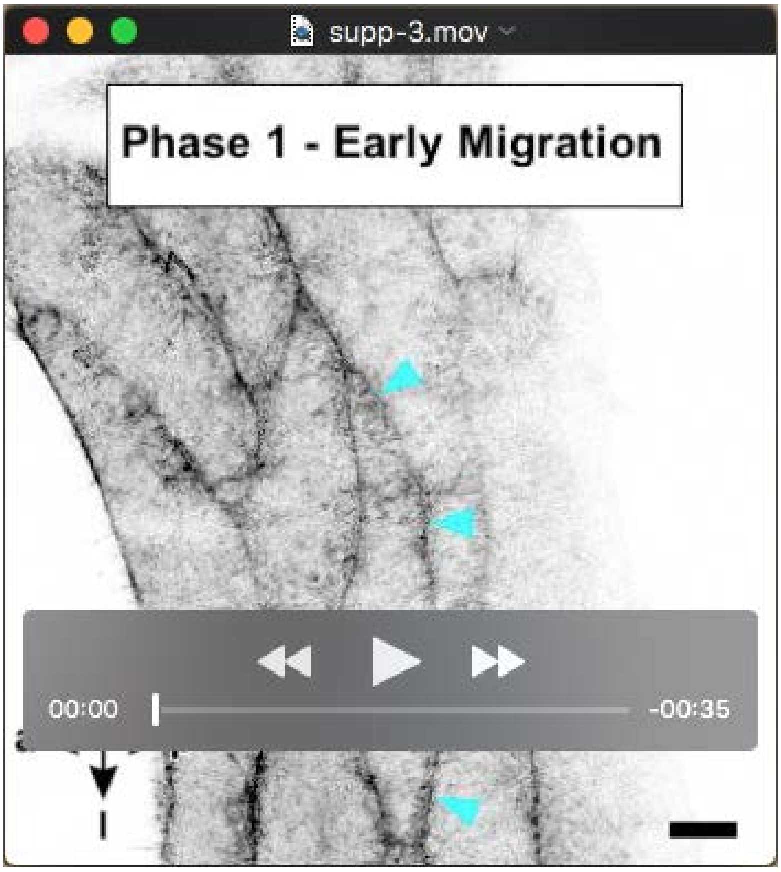

Movie 2. Different phases of LEC behaviour. Confocal micrographs of LECs labelled with GMA-GFP. During early migration (phase 1), LECs migrate without showing pulsed contractions. Merely flickering of apical activity is visible. During late migration (phase 2), LEC shows two actin foci alternating in their back (red dots). During constriction (phase 3), LEC shows an individual central actin focus (yellow dot). Cyan arrowheads, lamellipodium; hb, histoblasts; scale bar, 10 µm; a, anterior; p, posterior; m, medial (corresponds to dorsal); l, lateral (corresponds to ventral).

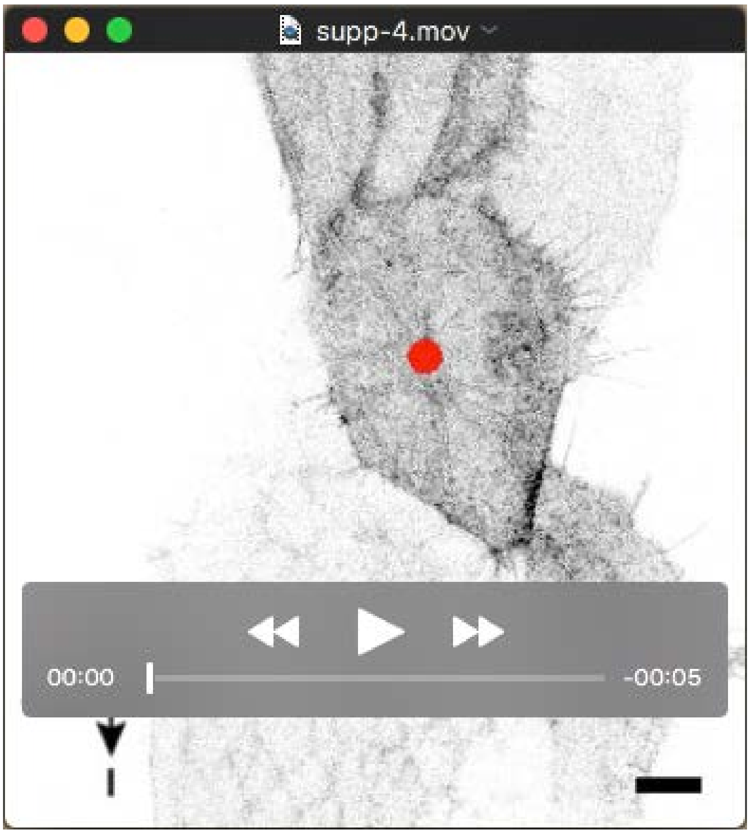

Movie 3. During dorsal repolarisation of a LEC, pulsatile activity reorganises to the back of the dorsally-migrating cell. Confocal micrographs of LECs labelled with GMA-GFP. Before repolarisation, actin focus is in the cell centre (1st red dot). After repolarisation, actin focus is found in the back of the cell (2nd red dot). hb, histoblasts; scale bar, 10 µm; a, anterior; p, posterior; m, medial (corresponds to dorsal); l, lateral (corresponds to ventral).

Journal of Cell Science • Supplementary information

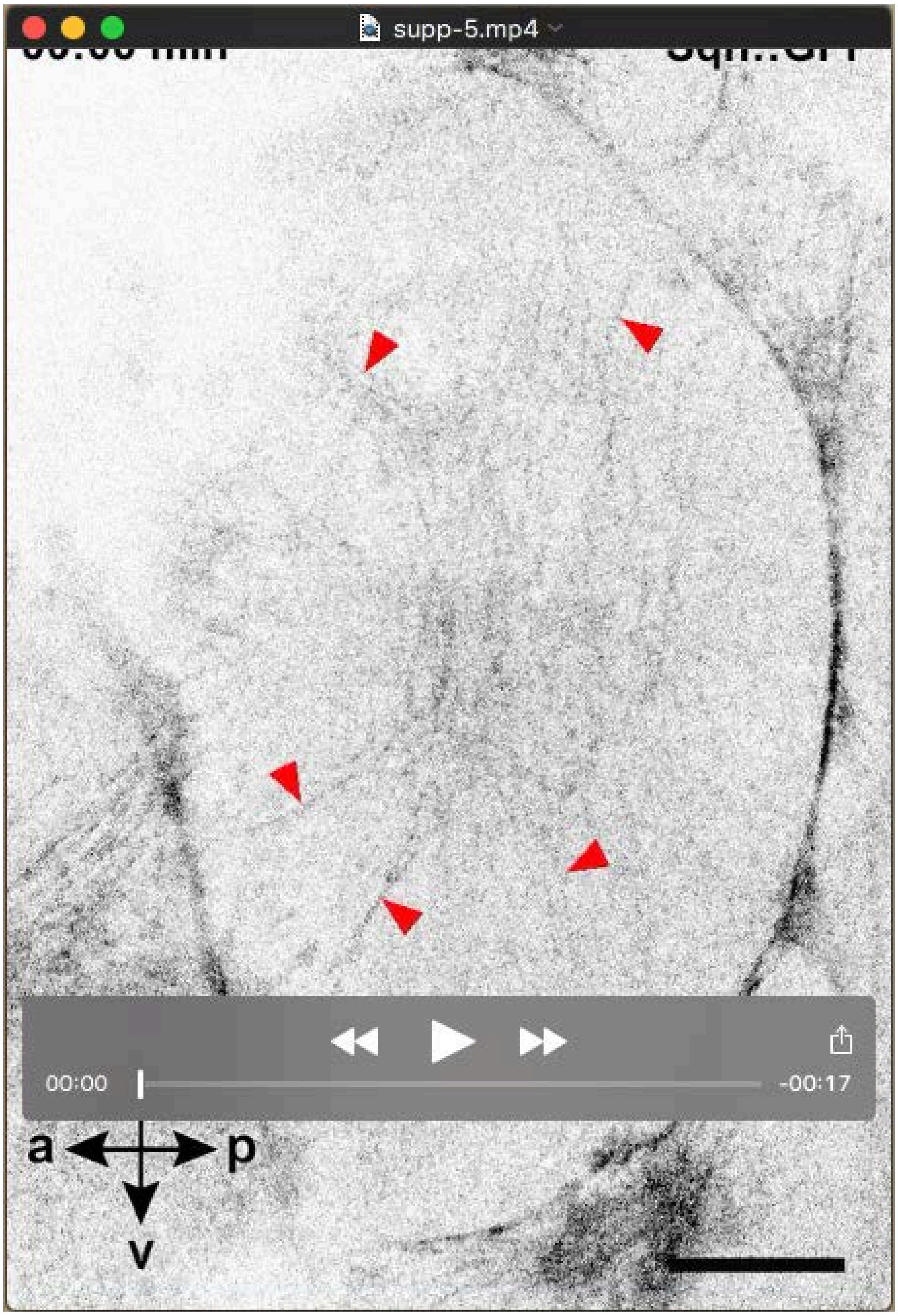

Movie 4. Cytoskeletal architecture and pulsatile activity of a constricting LEC. Sqh::GFP labels actin foci, actin bundles and cell-cell interfaces during constriction. Radially organised actin bundles (red arrowheads) connect the apicomedial network to the cell cortex. The contractile event begins in the cell periphery and then moves towards the cell centre. Red ellipsoids indicate movement of fluorescence signal towards the cell centre. Red dot shows actin focus at full contraction. Scale bar, 10 µm; a, anterior; p, posterior; m, medial (corresponds to dorsal); l, lateral (corresponds to ventral).

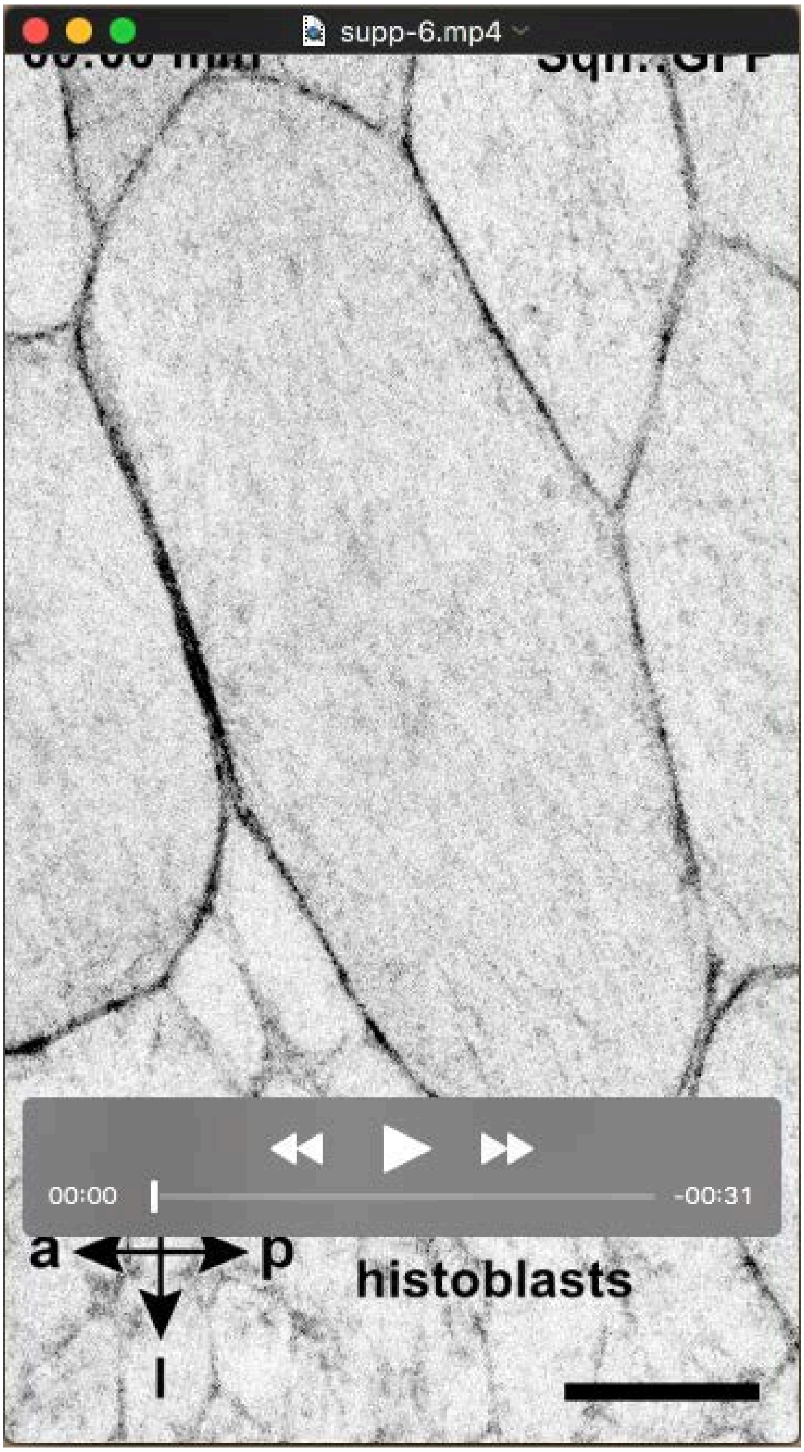

Movie 5. Constrictive behaviour of boundary LEC at the beginning of histoblast nest expansion, early during morphogenesis. Sqh::GFP labels cell-cell interfaces. Also, some diffuse labelling in the apical cell area, but no actin foci are visible. LEC constricts over time. Scale bar, 10 µm; a, anterior; p, posterior; m, medial (corresponds to dorsal); l, lateral (corresponds to ventral).

Journal of Cell Science • Supplementary information

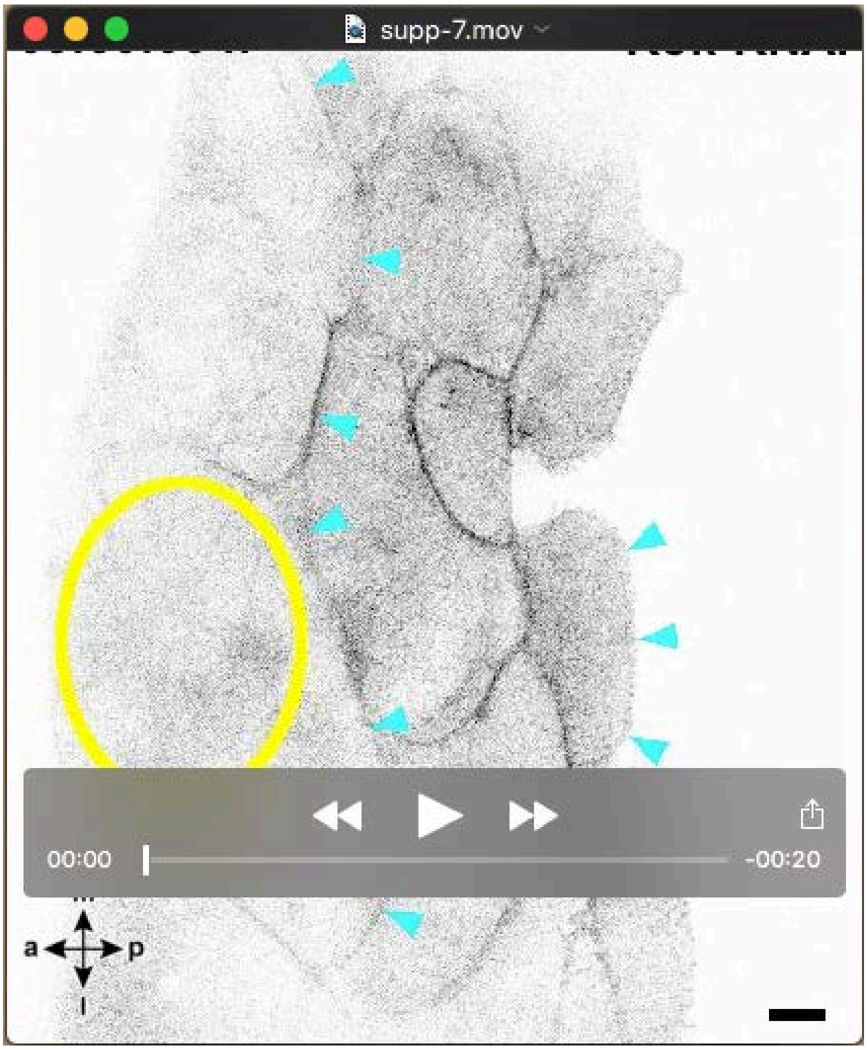

Movie 6. Overview movie of Rok-RNAi LECs showing impaired contractile behaviour and actin flows in the back of the cells. Overview confocal micrographs of LECs during late migration and constriction, F-actin labelled with GMA-GFP. LECs generate a lamellipodium in the front (cyan arrowheads) and show an apicomedial network, which does not generate clear foci, but only shows some diffuse activity (e.g. in area highlighted by yellow ellipsoid). Some cells create contractile flows in their back (cyan asterisks). LECs constrict and delaminate eventually (red arrow). Scale bar, 10 μm; a, anterior; p, posterior; m, medial (corresponds to dorsal); l, lateral (corresponds to ventral).

Journal of Cell Science • Supplementary information

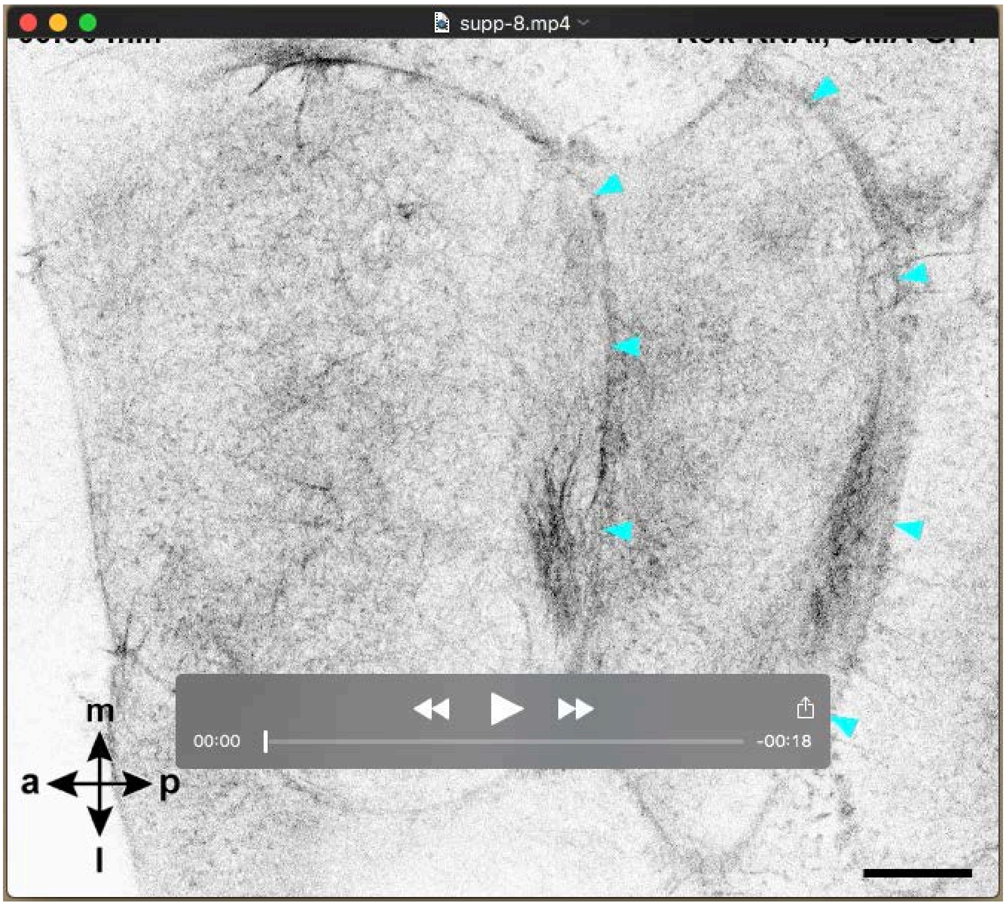

Movie 7. Two Rok-RNAi LECs showing impaired contractile behaviour and actin flows in the back of their neighbours. Detail confocal micrographs of two LECs during late migration, F-actin labelled with GMA-GFP. Cells generate lamellipodium in the front (cyan arrowheads) and show a not very dynamic apicomedial network, which does not generate clear foci, but shows only some diffuse activity. The cell on the right and its right-hand neighbour create contractile flows in their back (cyan asterisks), which lie underneath the lamellipodium of their left-hand neighbour (orange line highlights the overlapping region of the cell on the right). Scale bar, 10 µm; a, anterior; p, posterior; m, medial (corresponds to dorsal); l, lateral (corresponds to ventral).

Journal of Cell Science • Supplementary information

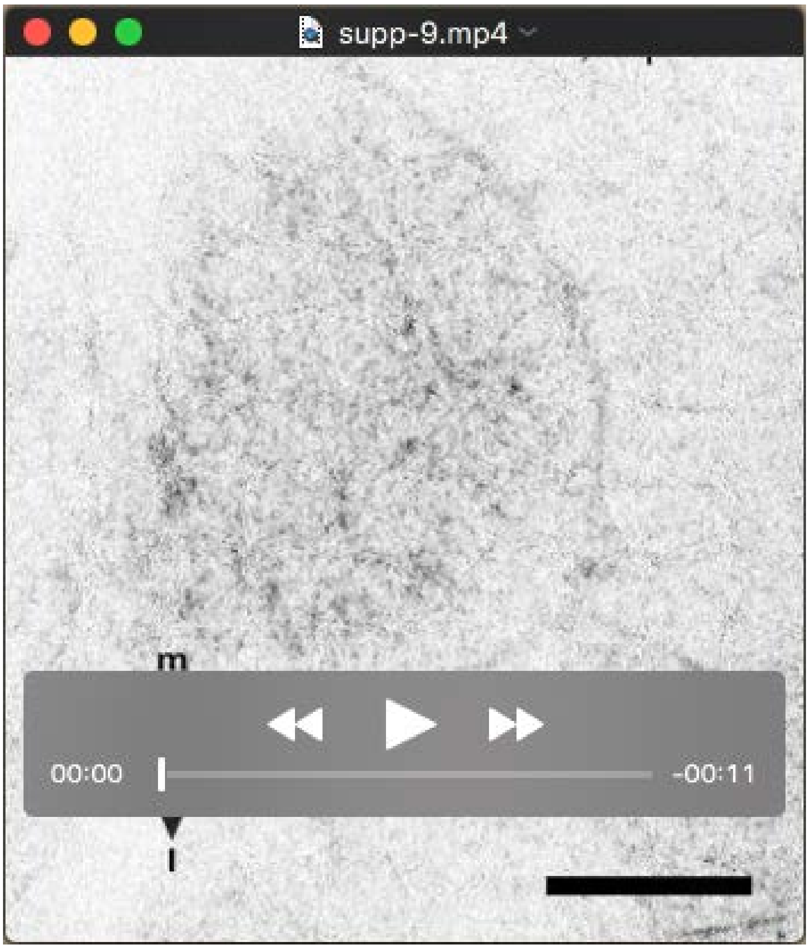

Movie 8. Rok-RNAi LEC labelled with Sqh::GFP showing impaired contractile behaviour during constriction. Confocal micrographs. Cell merely shows diffuse Sqh::GFP activity compared to controls (Movie 4). Apicomedial cytoskeletal activity resembles that of early boundary LECs (Movie 5). Scale bar, 10 μm; a, anterior; p, posterior; m, medial (corresponds to dorsal); l, lateral (corresponds to ventral).

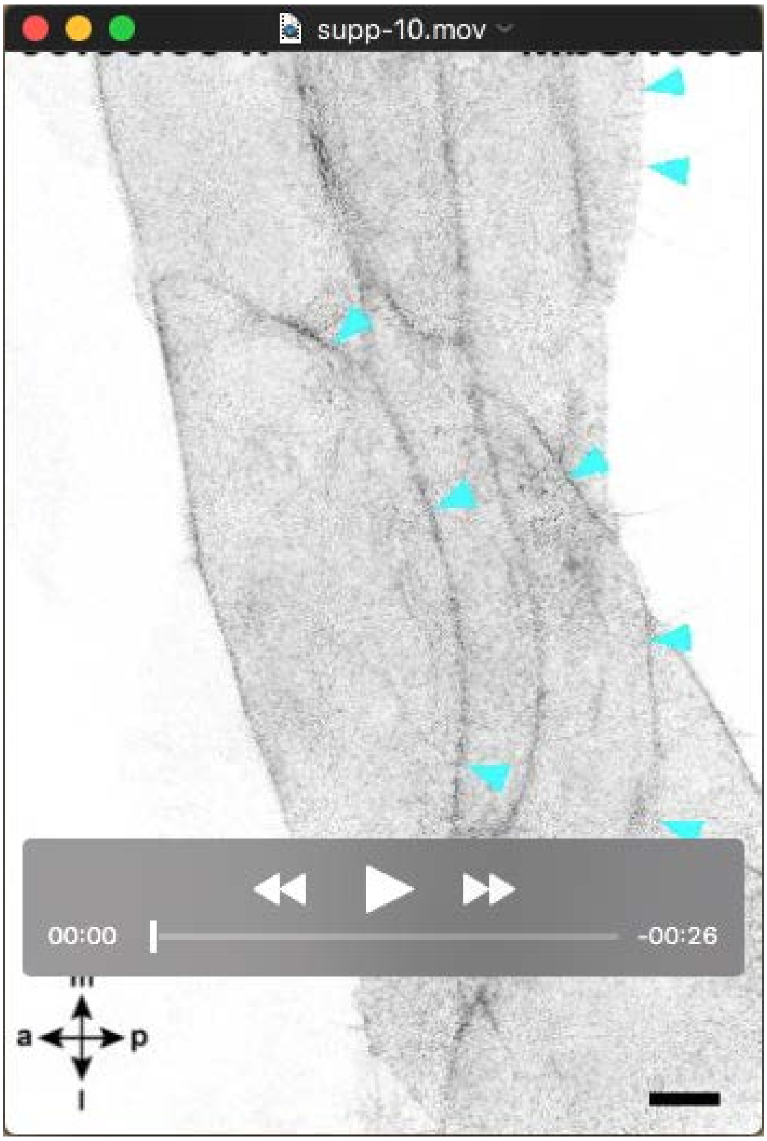

Movie 9. Overview of MbsN300 overexpressing LECs showing impaired contractile behaviour and actin flows in their back. Overview confocal micrographs of LECs during late migration and constriction, F-actin labelled with GMA-GFP. Cells generate lamellipodium in the front (cyan arrowheads) and show an apicomedial network, which does not generate clear foci, but only shows some diffuse activity (e.g. in area highlighted by yellow ellipsoid). Some cells create contractile flows in their back (cyan asterisks). LECs constrict and delaminate eventually (red arrow). Hb, histoblasts. Scale bar, 10 µm; a, anterior; p, posterior; m, medial (corresponds to dorsal); l, lateral (corresponds to ventral).

Journal of Cell Science • Supplementary information

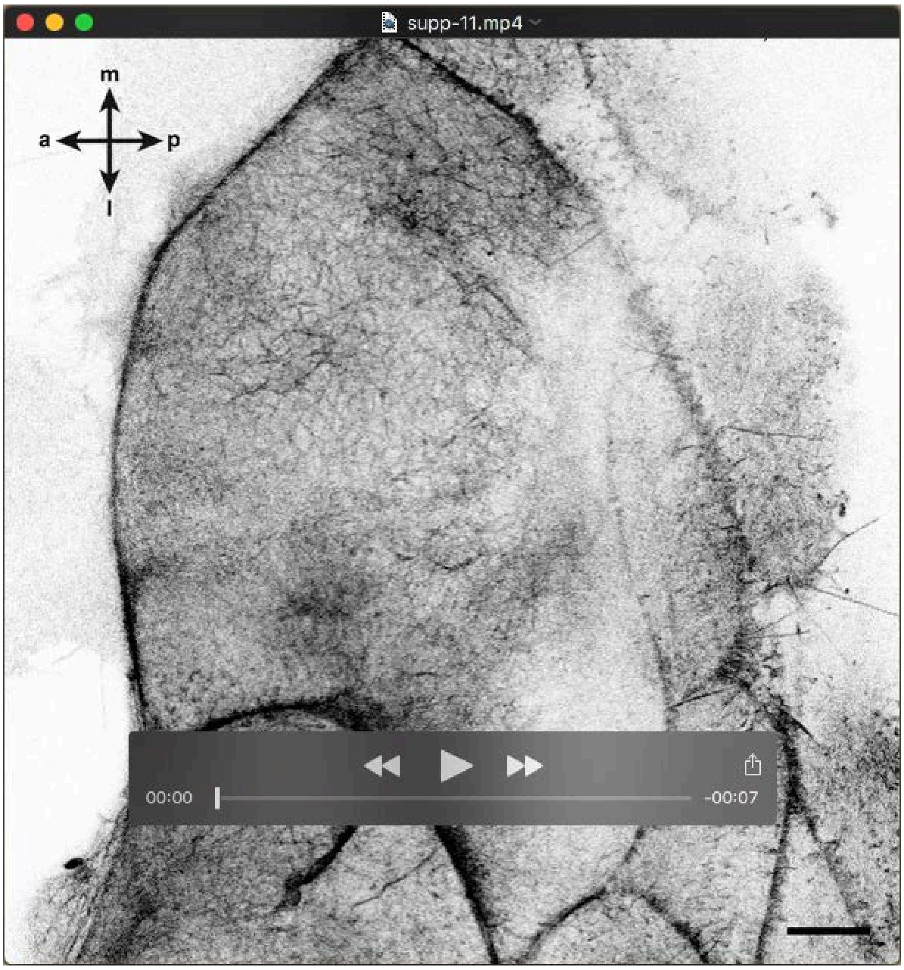

Movie 10. MbsN300 overexpressing LEC showing impaired contractile behaviour. Detail confocal micrographs of a LEC during early constriction. F-actin labelled with GMA-GFP. Cell only shows rudimentary pulsatile behaviour of a not very dynamic apicomedial network, which does not generate clear foci, but only shows some diffuse activity, which moves around the apicomedial network. Scale bar, 10 μm; a, anterior; p, posterior; m, medial (corresponds to dorsal); l, lateral (corresponds to ventral).

Journal of Cell Science • Supplementary information

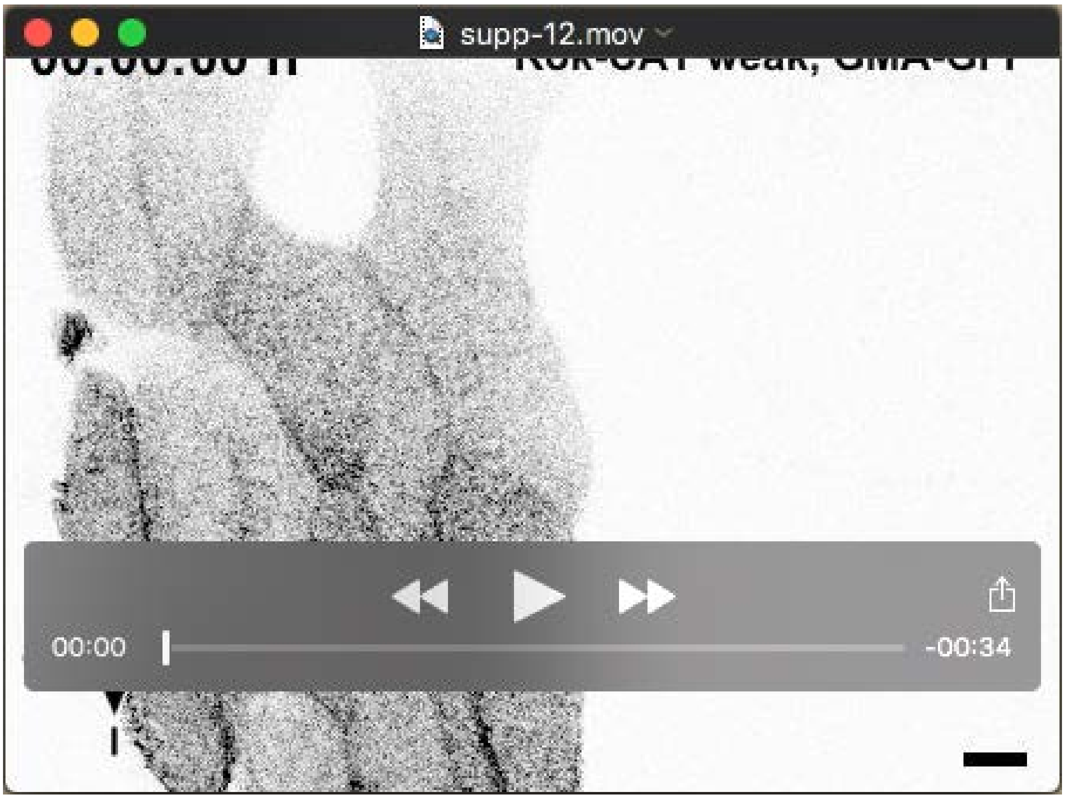

Movie 11. Rok-CAT overexpression in LECs at 25ºC, 'weak' phenotype. F-actin labelled with GMA-GFP. Beginning of morphogenesis, migration and constriction visible. Constricting LECs show high levels of cortical actin (blue arrowheads). LECs migrate, showing lamellipodia (cyan arrowheads) and blebbing (pink arrowheads). Some LECs show pulsatile behaviour (red dot). Scale bar, 10 µm; a, anterior; p, posterior; m, medial (corresponds to dorsal); l, lateral (corresponds to ventral).

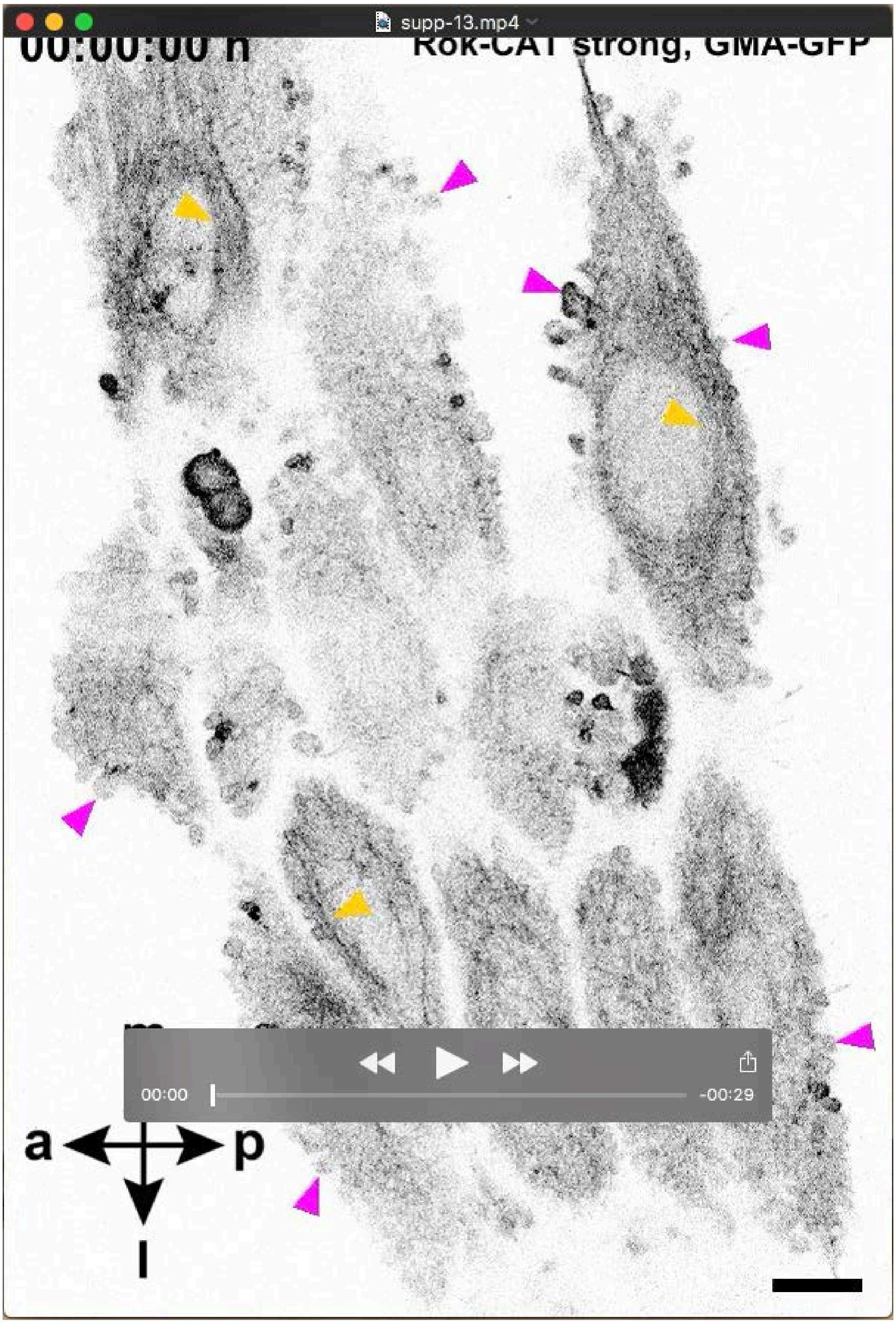

Movie 12. Rok-CAT overexpression in LECs at 29ºC, 'strong' phenotype. F-actin labelled with GMA-GFP. LECs are merely constricting, showing extensive blebbing (pink arrowheads) and cortical actin bundles (orange arrowheads). No pulsatile behaviour visible. Eventually, cells delaminate (red arrow). Scale bar, 10 µm; a, anterior; p, posterior; m, medial (corresponds to dorsal); l, lateral (corresponds to ventral).

Journal of Cell Science • Supplementary information

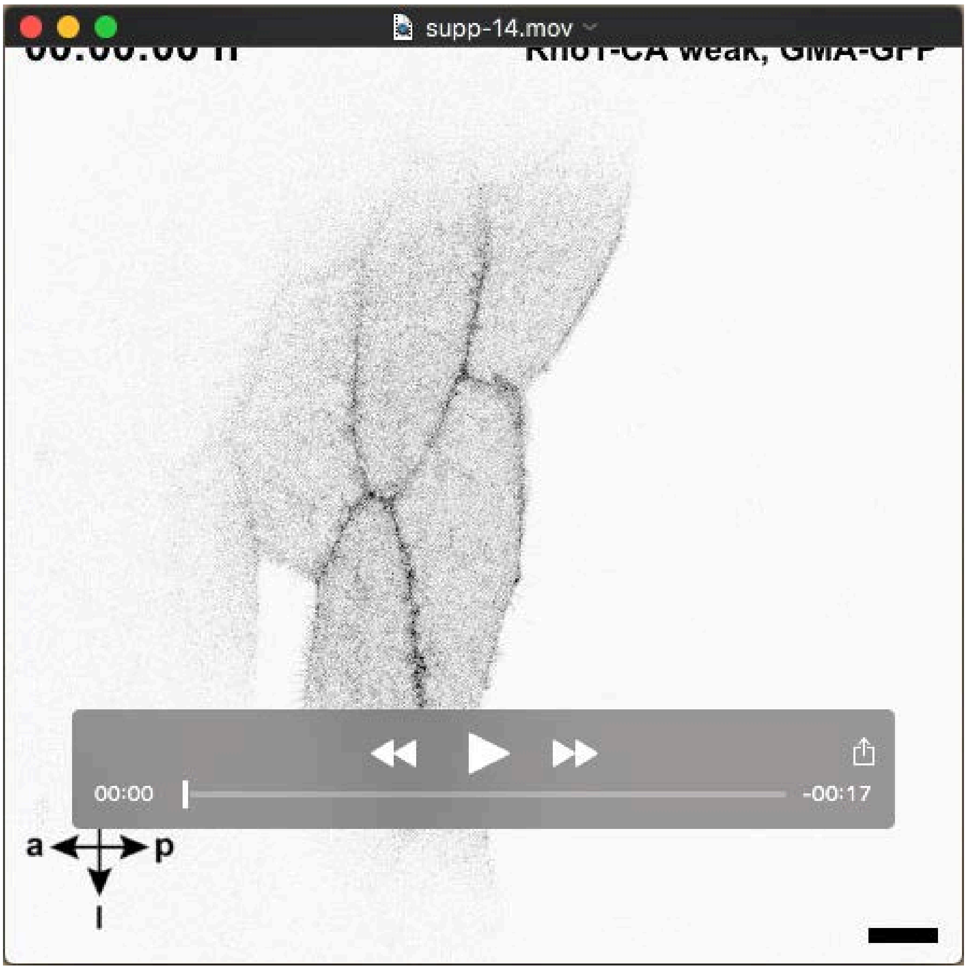

Movie 13. Rho1-CA overexpression in LECs, ‘migrating’ phenotype. F-actin labelled with GMA-GFP. Beginning of morphogenesis, migration and constriction visible. LECs migrate, showing lamellipodia (cyan arrowheads); constricting LECs show blebbing (pink arrowheads). No pulsatile behaviour visible. Scale bar, 10 µm; a, anterior; p, posterior; m, medial (corresponds to dorsal); l, lateral (corresponds to ventral).

Journal of Cell Science • Supplementary information

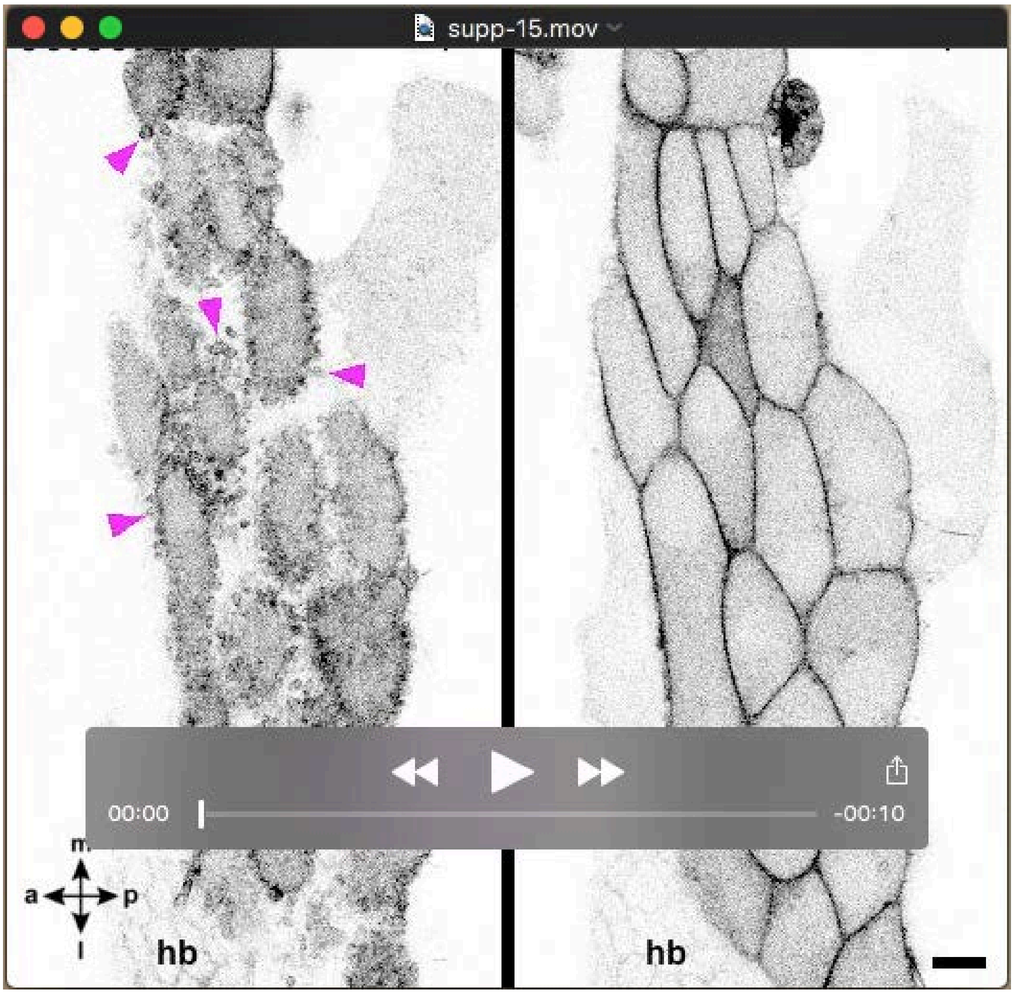

Movie 14. Rho1-CA overexpression in LECs, 'non-migrating' phenotype. Apical (left) and apicolateral (right) *z*-slice shown. F-actin labelled with GMA-GFP. LECs are merely constricting, showing extensive blebbing (pink arrowheads). No pulsatile behaviour visible. Eventually, cells delaminate (yellow arrow). Scale bar, 10 µm; a, anterior; p, posterior; m, medial (corresponds to dorsal); l, lateral (corresponds to ventral).

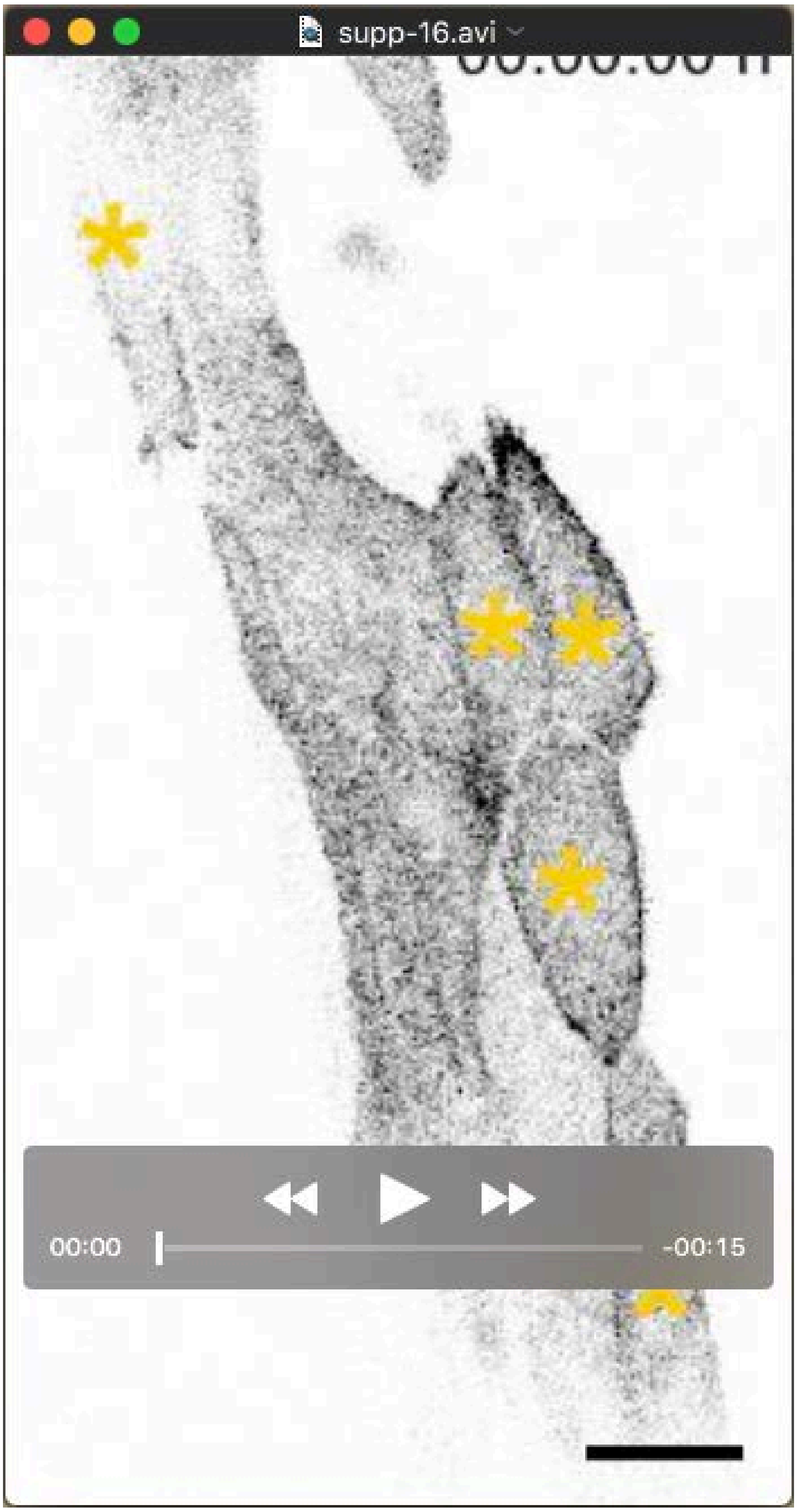

Movie 15. Rho1 overexpression in LECs. F-actin labelled with GMA-GFP. Orange asterisks indicate LECs that cycle between (1) the presence of apicomedial actin and (2) the absence of apicomedial actin but increased junctional cortical actin and blebbing. Pink arrowhead highlights LEC which is blebbing and shows junctional cortical actin. Scale bar, 20 µm. Anterior is to the left.
